# A Family of Bisnaphthyl C_2_-Symmetric and Asymmetric Clefts: Synthesis, Solid-State Structure, and Calculation of the Interplanar Angle

**DOI:** 10.1021/acs.joc.2c03002

**Published:** 2023-02-28

**Authors:** Gazalah S. Mohammed Elgadi, Mark R. J. Elsegood, Miheal Patel, Paulo. A. Netz, Tiago E. de Oliveira, Marc C. Kimber

**Affiliations:** † The Department of Chemistry, School of Science, Loughborough University, LE11 3TU Loughborough, U.K.; ‡ Grupo de Química Teórica, Instituto de Química, Universidade Federal do Rio Grande do Sul, Porto Alegre, RS CEP 91501-970, Brazil; § Departmento de Farmacociências, Universidade Federal de Ciências de Saúde Porto Alegre, Porto Alegre, RS 90050-170, Brazil

## Abstract

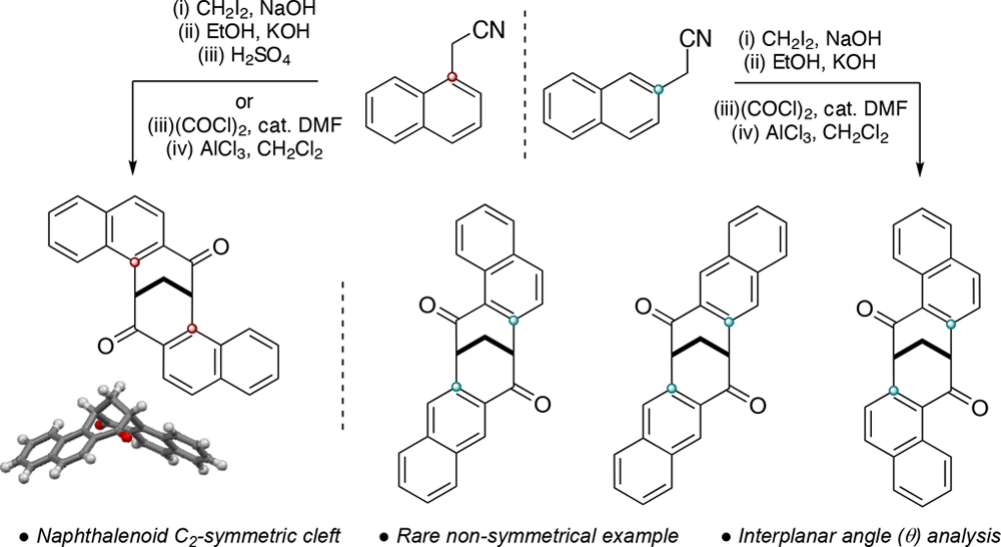

The synthesis of a new family of naphthalenoid C_2_-symmetric clefts has been realized through a four-step synthetic sequence giving three C_2_-symmetric clefts and a rare nonsymmetric example. Subsequently, stereoselective reduction of the carbonyl groups at C-8 and C-16 then provides cleft molecules with hydrogen bonding potential. Using single-crystal X-ray and computational analysis, the cleft angle of the dione has been determined.

Dibenzobicyclo[*b*,*f*][3.3.1]nona-5a,6a-diene-6,12-dione (**1**) is a C_2_-symmetric molecule with a distinctive 3D cleft shape, a consequence of three contiguous bridgehead sp^3^ hybridized carbons that “fix” the conformation of the molecule (Scheme 1A).^1,2^

**Scheme 1:**
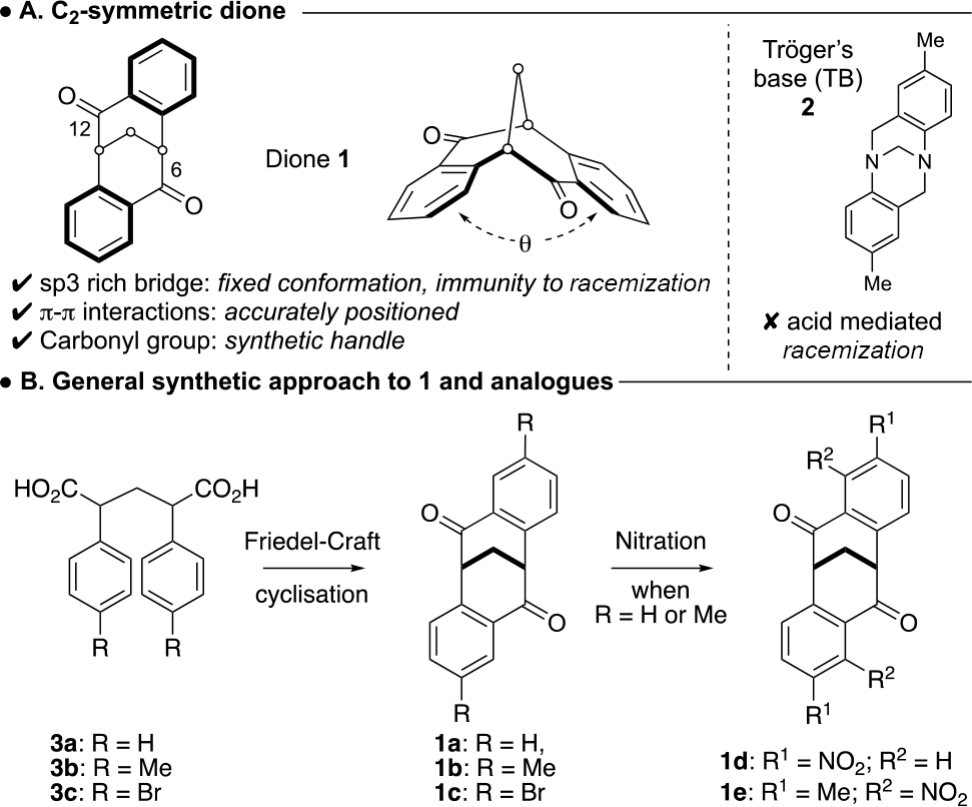
(A) Dibenzobicyclo[*b*,*f*][3.3.1]nona-5a,6a,diene-6,12-dione (**1**) and Tröger’s Base (TB, **2**) and (B) Synthesis of **1** and Analogues

The C_2_-symmetric axis, together with its unique topography, provides a unique chiral cavity that can be exploited in supramolecular interactions.^3−5^ Unlike its structural equivalent Trögers base (TB, **2**),^6,7^ its carbocyclic skeleton provides immunity to racemization in acidic media while also offering extra functionality through the two carbonyl groups at C-6 and C-12, respectively. However, it must be emphasized that TB (**2**) has been extensively functionalized^7^ and is an exemplar chiral cleft for dione **1** to aspire to in terms of flexibility and synthetic potential.

The synthesis of **1**, and its analogues, is achieved through a double Friedel–Crafts cyclization of a diacid **3** (Scheme 1B). This route provides **1a**, as well as the methyl- (**1b**) and bromo-analogue (**1c**) derivatives.^8^ Alternatively, analogues of **1** can be accessed through direct chemoselective manipulation of the two aromatic rings (**1d** and **1e**).^9^ Functionalization of the two aromatic rings is important, as it directly affects the cleft angle (θ) and can provide predictability in their positioning. Importantly, the two carbonyl groups at positions C-6 and C-12 of **1** can be stereoselectively reduced, providing anchor points for the introduction of host–guest recognition features.^10−16^

However, in contrast to its structural equivalent TB (**2**), there are very few examples of **1** possessing extended aromaticity,^17^ even though this structural feature is known to enhance host–guest interactions and physicochemical properties.^18^ The importance of extended aromaticity in naphthalenoid TB analogues has led to the development of molecular tweezers,^19−21^ analogues that exhibit high specific rotations^22^ and ligands that interact *selectively* with biomolecules such as DNA.^23−25^ Consequently, presented with the benefits of extended aromaticity together with the structural deficiencies of TB, the synthesis of naphthalenoid analogues of **1** would be welcomed.

Therefore, in this disclosure we report the synthesis of a naphthyl family of carbocyclic dione clefts. Their 3D structure is fully characterized and supported by single-crystal X-ray crystallography, which then permits an analysis of the cleft angles of each naphthalenoid isomer.

Three C_2_-symmetric naphthalenoid clefts were targeted for synthesis (**5a**–**c**), all of which possess increased aromatic surface compared with the parent cleft **1** (Scheme 2). The synthesis of **5a** is conceivable by a Friedel–Crafts cyclization on diacid **4a** through the 2-naphthyl carbon. The clefts **5b** and **5c** can also be synthesized through a Friedel–Crafts cyclization on diacid **4b**, with **5b** being accessed through the 1-naphthyl carbon and **5c** through the 3-naphthyl carbon.

**Scheme 2:**
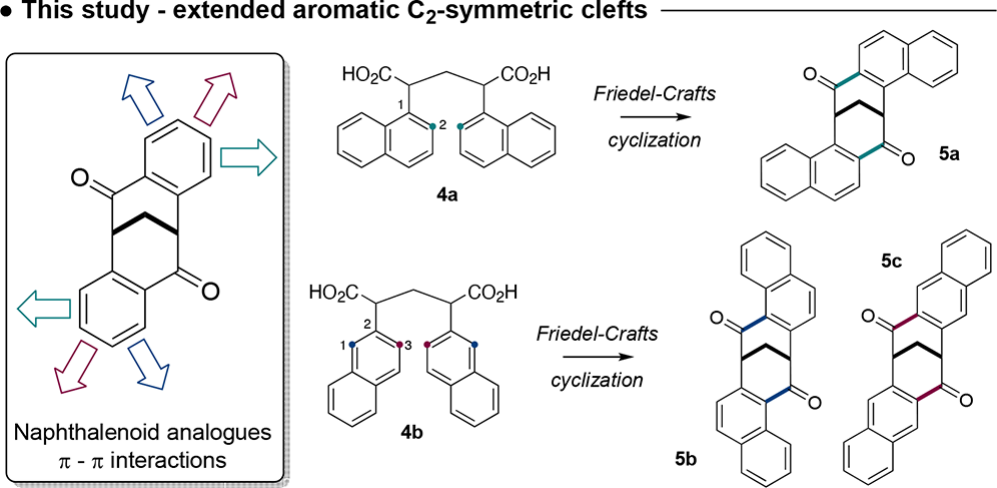
Targeted Naphthalenoid Carbocyclic Clefts and Synthetic Approach

The synthesis of the first naphthyl ligand **5a** began with 1-acetonitrilenaphthalene (**6a**) and diiodomethane using an adapted procedure of Tatemitsu (Scheme 3).^1^ Condensation of **6a** and CH_2_I_2_ in the presence of potassium hydroxide provided a crude dinitrile that was subsequently hydrolyzed to give **4a**, as a mixture of its *meso* and (±)-isomers, in a 51% yield over these two steps. Treatment of this mixture with concentrated sulfuric acid then effected a double Friedel–Crafts cyclization, giving (±)-**5a** in an isolated yield of 4% (conditions A). The structure of the C_2_-symmetric naphthalenoid dione (±)-**5a** was fully established through ^1^H and ^13^C NMR spectroscopy and further supported by single-crystal X-ray analysis, with the molecular structure also shown in Scheme 3.

**Scheme 3:**
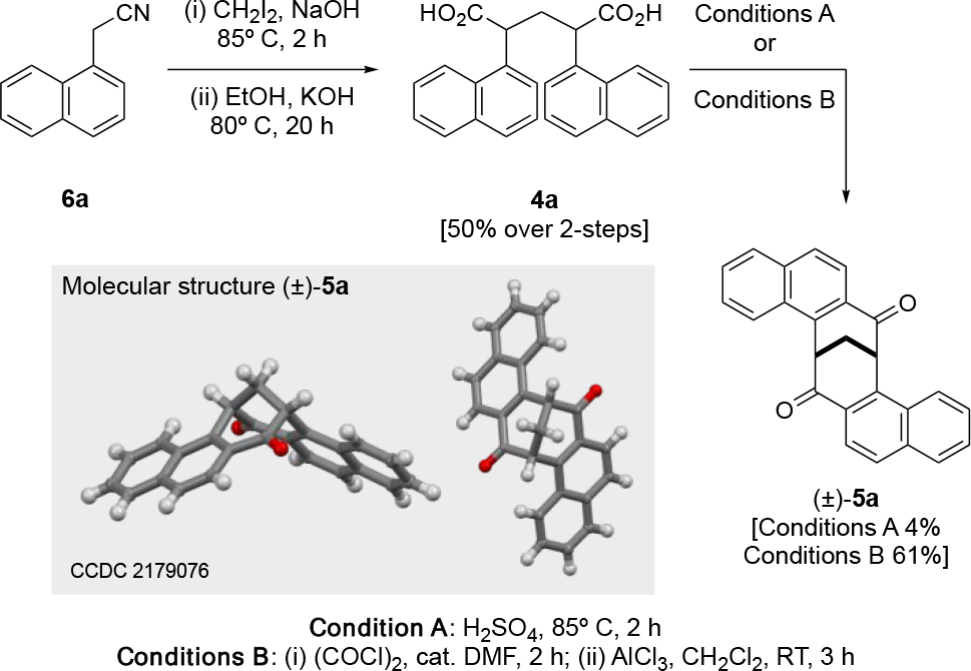
Synthesis of Naphthalenoid Cleft **5a** from 1-Acetonitrilenaphthalene **6a**

The yield of (±)-**5a** using conditions A was very low and significantly depressed compared with the cyclization of diacid **3a**, which provides the parent dione **1a** in *ca*. 40% isolated yield.^9^ We suspected the depressed yield in the formation of (±)-**5a** was due to the sulfonation of the naphthyl ring under the sulfuric acid-mediated Friedel–Craft cyclization conditions. We therefore sought an alternate method that would circumvent the sulfonation of either the starting diacid **4a**, the product dione (±)-**5a**, or both. An improvement in the synthesis was found by the treatment of the diacid mixture **4a** with (COCl)_2_ to provide the diacid chloride, whose cyclization to the dione (±)-**5a** could then be effected with AlCl_3_ (conditions B). This reaction sequence provided (±)-**5a** in an improved isolated yield of 61%. The physical data of (±)-**5a** using these improved conditions were identical to those of the product obtained using conditions A.

The naphthalenoid dione targets **5b** and **5c** were synthesized from 2-acetonitrilenaphthalene (**6b**) (Scheme 4). Again, condensation of **6b** and CH_2_I_2_ in the presence of potassium hydroxide, followed by immediate saponification of the mixture, gave **4b** in a 38% yield over two steps. The cyclization of this diacid mixture using concentrated sulfuric acid (condition A) yielded one identifiable product in an isolated yielded of 6%. Subsequently, ^1^H and ^13^C NMR analysis showed this product to be the naphthalenoid dione (±)-**5b**.^17^

**Scheme 4:**
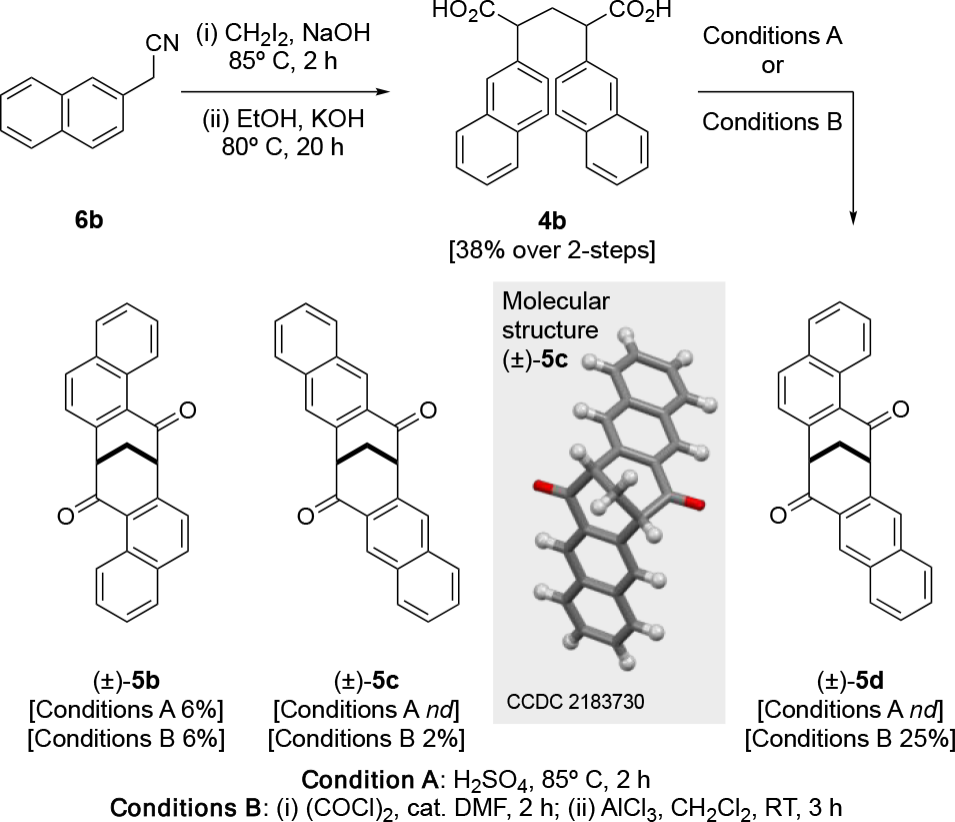
Synthesis of Naphthalenoid clefts **5b**–**d** from 2-Acetonitrilenaphthalene **6b**

Employing conditions B to achieve the cyclization of the diacid **6b** provided three products after purification. Detailed ^1^H and ^13^C NMR analysis signified the formation two C_2_-symmetric naphthalenoid diones (±)-**5b** and (±)-**5c**, which were isolated in 6% and 2% yields, respectively, with a remaining unknown product being isolated in a 26% yield.

Compared with (±)-**5a**–**c**, the ^1^H NMR spectrum of the remaining unidentified product was atypical. Characteristically, the bridge protons of parent dione **1** and the naphthalenoid C_2_-symmetrical diones (±)-**5a**–**c** present as a triplet, typically in the region of δ 3.30–2.95 ppm. However, the ^1^H NMR spectrum of the unidentified product exhibited increased complexity in this region, signifying that the product was no longer C_2_-symmetric. Further analysis indicated that the bridge protons were presenting as diastereotopic, at δ 3.21 and 3.05 ppm, respectively; this was further supported by the ^13^C NMR spectra. Therefore, we proposed the structure of the unknown product to be that shown for (±)-**5d**. We were able to obtain crystals for all three clefts (±)-**5b**–**d**, and this further supported our NMR analysis, confirming their proposed structures; the molecular structure of (±)-**5c** is shown in Scheme 4. The naphthalenoid dione (±)-**5d** is a very rare example of an unsymmetrical cleft within this family of cleft molecules and should find relevance with the wider family of TB naphthalenoid derivatives.^19−22,26^

Previously we have reported that the reduction of **1a** with NaBH_4_ provides a diol with two hydroxy groups that are ideally positioned for chiral recognition.^14,15^ Accordingly, the reduction of diones (±)-**5a** and (±)-**5b** was realized by treatment with methanolic NaBH_4_ providing diols (±)-**7a** and (±)-**7b** in 51% and 47% yields, respectively (Scheme 5).

**Scheme 5:**
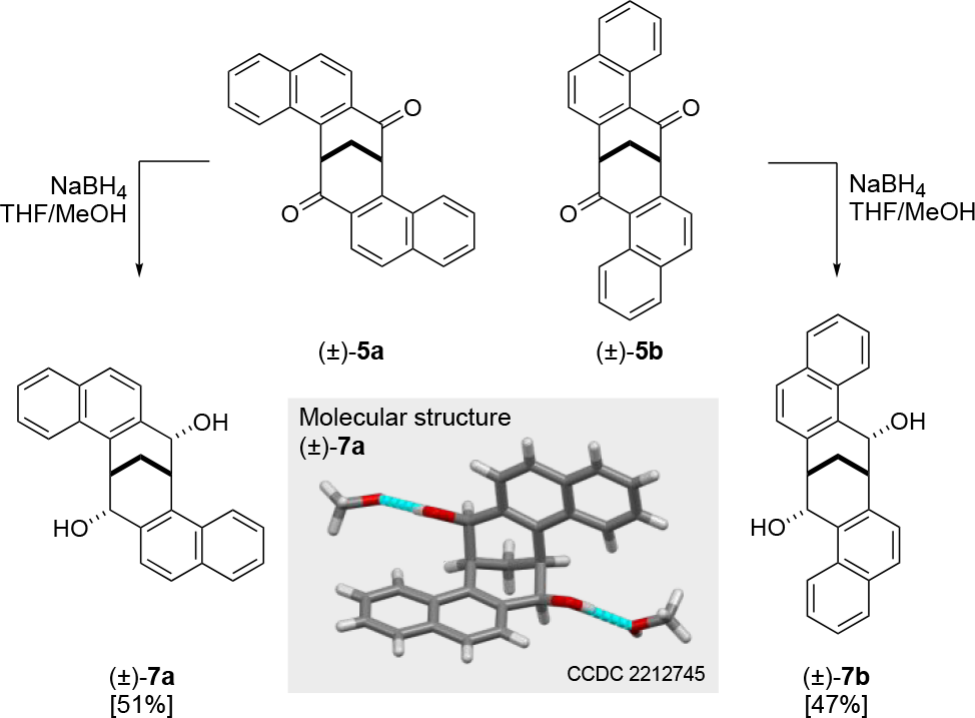
Stereoselective Reduction Providing Diols (±)-**7a** and (±)-**7b**

Detailed ^1^H and ^13^C NMR analysis of (±)-**7a** and (±)-**7b** indicated that, analogous with **1**, the reduction is entirely stereospecific, with each hydroxyl group being directed into the cavity of the cleft. This observation was further confirmed by the single-crystal X-ray analysis performed on (±)-**7a**, whose molecular structure is shown in Scheme 5. This X-ray analysis also shows H-bonding of the diol with two molecules of methanol, therefore demonstrating the potential for this cleft class.

Pleasingly, the results above enabled an analysis of the interplanar “cleft” angles (θ) for each of the four diones (±)-**5a**–**d** and the diol (±)-**7a** (Table 1). This angle was calculated between the two aromatic rings directly attached to the saturated cleft; furthermore, this measure maybe useful in the rationale design of host–guest interactions within the cavity of the cleft. The interplanar “cleft” angle for naphthalenoid dione (±)-**5a**, was considerably higher, 105.2°, compared with those of diones (±)-**5b**–**d**. These later diones exhibited cleft angles in the range of 91–94° approximating that of TB (**2**).^27^ The flexibility in the interplanar angle of this dione cleft family has been observed in previous studies,^8,9^ with substitution on the benzene rings of **1** greatly affecting the interplanar angle. Electron-donating groups on the aryl ring of **1** have been shown to increase the interplanar angle, with the dimethyl ligand **1a** exhibiting the largest angle (104.3° or 101.7°) to date; this value is now surpassed by (±)-**5a**. The cleft angles were also determined from the computational optimized structures of **5a**–**d** and **7a**.^28^ Apart from **5a**, there are discrepancies in these cleft angle values for **5b**–**d** and **7a**, and this most likely reflects the defined crystal packing and has been observed in the X-ray crystallographic data for related analogues of **1**.^14,15^

**Table 1:**
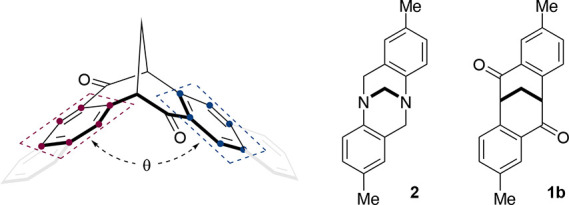
Interplanar Angle (θ) between Planes Calculated from the Two Aromatic Rings Directly Attached to the Cleft

	interplanar angle (θ) (°)^a^	
cleft molecule	X-ray structure	calculated
(±)-**5a**	105.2	107.4
(±)-**5b**	92.9	100.9
(±)-**5c**	91.3	103.8
(±)-**5d**	93.2	101.9
(±)-**7a**	90.7	103.7
**2** (TB)^b^	92.9, 97.4^c^	
**1b**^d^	104.3, 101.7^c^	

a The interplanar angle was calculated between the two aromatic rings directly attached to the saturated cleft.

b Ref (26).

c Two molecules in the asymmetric unit.

d Ref (9).

The mechanism for the formation of dione (±)-**5a** mirrors that of **1**, occuring through initial oxonium formation to give **I**, which undergoes a Friedel–Crafts cyclization with the 2-naphthyl carbon to give intermediate **II**; this cyclizes again, providing the bicyclic dione (±)-**5a** (Scheme 6A). Note that only (±)-**I** undergoes cyclization and that its *meso* isomer cannot due to geometrical constraints.

**Scheme 6:**
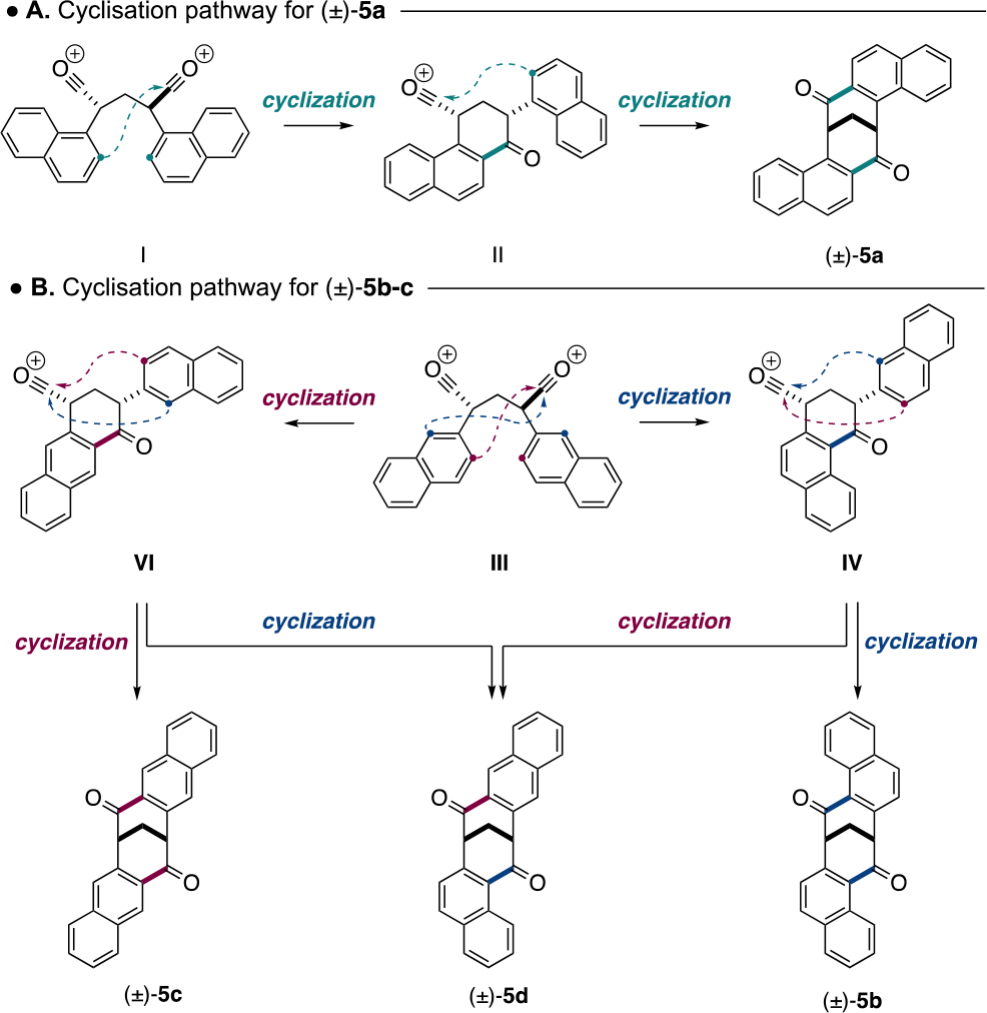
Cyclization Pathway for the Formation of (±)-**5a**–**d**

The formation of (±)-**5b**–**d** is more complicated given there are two potential nucleophilic sites on the oxonium **III**: the 1-naphthyl carbon and 3-naphthyl carbon, respectively (Scheme 6B). Cyclization of oxonium **III** via the 1-naphthyl carbon provides the oxonium intermediate **IV**, which in turn can cyclize via two subsequent pathways. Cyclization via the second 1-naphthyl carbon provides the C_2_-symmetric dione (±)-**5b**, whereas cyclization via the 3-naphthyl carbon provides the unsymmetrical dione (±)-**5d**. Alternatively, cyclization of oxonium **III** via the 3-naphthyl carbon provides oxonium intermediate **VI**. Again, this can cyclize via two pathways, with cyclization via the second 3-naphthyl carbon providing the C_2_-symmetric dione (±)-**5c** and cyclization via the 1-naphthyl carbon giving the unsymmetrical dione (±)-**5d**.

To conclude, in this study we have synthesized a new series of naphthalenoid C_2_-symmetric clefts possessing extended aromaticity. Additionally, a rare unsymmetrical naphthalenoid cleft could be accessed. The single-crystal X-ray structure data for the four diones provided an analysis of the interplanar cleft angle, with dione **5a** exhibiting an angle of 105.2°, the largest yet reported for this family of cleft substrates. Given the structural advantages of dione **1**(29) (e.g., immunity to racemization and carbonyl functionalization) compared with TB, these new C_2_-symmetric cleft molecules hold potential as scaffolds, given the historical success of TB analogues in medicinal chemistry. There exists significant scope in further developing this family of ligands by synthesizing each of their enantiomers using existing chemistry^15^ and investigating the synthetic manipulation of the naphthyl rings.^9^ Additionally, we are currently assessing the interactions of (±)-**5a**–**d** with biomolecules such as ct-DNA and will report these results in due course.

## Data Availability

The data underlying this study are available in the published article and its Supporting Information

## Supporting Information Available

Experimental procedures, compound characterization, crystallographic data, computational tables, and copies of NMR spectra (PDF)
