# Supplementary material for: A Family of Bisnaphthyl C2-Symmetric and Asymmetric Clefts: Synthesis, Solid-State Structure, and Calculation of the Interplanar Angle
Source: J Org Chem. 2023 Feb 28;88(6):3965–9. doi: 10.1021/acs.joc.2c03002 (PMC10028689; doi:10.1021/acs.joc.2c03002)
Supplement: Supplementary file 1 — jo2c03002_si_001.pdf [file jo2c03002_si_001.pdf]

A family of *bis*-naphthyl C_2-symmetric and asymmetric clefts: synthesis, solid state structure and calculation of the interplanar angle

Gazalah S. Mohammed Elgadi,[a] Mark R. J. Elsegood,[a] Miheal Patel,[a] Paulo. A. Netz,[b] Tiago E. de Oliveira,[c] and Marc C. Kimber*[a]

[a] *The Department of Chemistry, School of Science, Loughborough University, Loughborough, LE11 3TU, UK.*

[b] *Grupo de Química Teórica, Instituto de Química, Universidade Federal do Rio Grande do Sul, Av. Bento Gonçalves 9500, CEP 91501-970, Porto Alegre, RS, Brazil*

[c] *Departmento de Farmacociências, Universidade Federal de Ciências de Saúde Porto Alegre, Porto Alegre 90050-170, Brazil.*

**M.C.Kimber@lboro.ac.uk*

Table of contents

Synthetic procedures for the preparation of (±)-**S1**, (±)-**5a-d,** (±)-**7a,b** S2

NMR spectra of prepared compounds S11

X-ray data for (±)-**5a-d** & (±)-**7a** S18

Interplanar angle calculations S35

References S41

Synthetic procedures for the preparation of (±)-**1**, (±)-**5a-d &** (±)-**7a,b**

General

Most of starting materials, reagents and solvents were purchased from Sigma-Aldrich and used as received. All reactions were carried out under an atmosphere of dry, oxygen-free nitrogen in oven-dried glassware. All chromatographic manipulations used silica gel as the adsorbent. Reactions were monitored by Thin Layer Chromatography (TLC) on aluminium backed plates with Merck Kiesel 60 F254 silica gel. TLCs were either visualized by UV radiation at a wavelength of 254 nm or stained by exposure to potassium permanganate aqueous solution, following by charring where appropriate. Purification by column chromatography was carried out using Merck Kiesel 60 H silica adsorbent in addition to combiflash NEXTGEN 100. Purification by Preparative Thin Layer Chromatography (Prep.TLC) was carried out on glass backed plates (20×20 cm) with silica gel GF. Melting points were recorded using an Electrothermal-IA 9100 melting point instrument. All infrared spectra were acquired in KBr disc unless otherwise indicated by using a Perkin-Elmer, spectrum 65 FT-IR spectrophotometer. The NMR spectra were recorded by using $CDCl_3$, $(CD_3)_2SO$ or $(CD_3)_2CO$ as solvents according to solubility of product. Both ^{1}H and ^{13}C were measured at 400 and 500 MHz using a Bruker DPX 400, 500 MHz spectrometer with chloroform-d$_1$ (δ 7.26, ^{1}H; δ 77.0, ^{13}C), dimethyl sulfoxide-d$_6$ (δ 2.50, ^{1}H; δ 39.5, ^{13}C) and acetone-d$_6$ (δ 2.09, ^{1}H; δ29.9, 206.7, ^{13}C) as internal standards unless otherwise indicated. HRMS were recorded using a Thermo Scientific Exactive Orbitrap mass spectrometer. Diffraction data for the x ray structures was collected on a Bruker APEX 2 CCD area detector with a graphite monochromator. *SHELXT* (Sheldrick, 2015). *SHELXL2018/3* (Sheldrick, 2018) programmes were used to resolve the x-ray structures.

Preparation of parent dibenzobicyclo-[*b,f*][3.3.1]-nona-5a,6a-diene-6,12-dione (±)-**S1**[1]

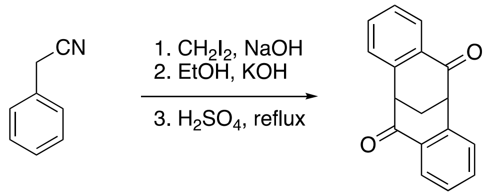

(±)-**S1**

To a mixture of phenylacetonitrile (2.30 mL, 20.00 mmol) and diiodomethane (0.80 mL, 10.00 mmol) was added finely powdered NaOH (0.79 g, 20.00 mmol). The resulting reaction mixture was stirred for 2 h at 145 °C, cooled to room temperature and a 1:1 mixture of water/ diethyl ether (5 mL) added. The reaction mixture was poured into water (10 mL) and the aqueous layer was extracted with diethyl ether (3 × 5 mL). The combined ethereal layers were dried over anhydrous $MgSO_4$, filtered, and the solvent removed under reduced pressure to give crude (±)- and *meso*-2,4-diphenylpentanedinitrile as a dark brown oil (3.47 g, 14.10 mmol, 70%). To a solution of crude dinitrile (3.47 g, 14.00 mmol) in ethanol (35 mL) was added a mixture of KOH (10.20 g, 181.80 mmol) and water (38 mL). The resulting reaction mixture was stirred at reflux temperature 80 °C for 20 h, cooled to room temperature, and the ethanol removed under reduced pressure. The aqueous layer was washed with diethyl ether (4 × 10 mL), acidified with concentrated hydrochloric acid, and extracted with ethyl acetate (3 × 15 mL). The organic layers were washed with water (2 × 10mL) and dried over anhydrous $MgSO_4$, filtered and the solvent then removed under reduced pressure to give crude (±)- and *meso*-2,4-diphenylpentanedioic acids as a brown oily-residue (3.25 g, 11.40 mmol, 81%). The crude mixture of (±)-and *meso*-2, 4-diphenylpentanedioic acids (3.25 g, 11.40 mmol) were dissolved in concentrated sulphuric acid (6.50 mL) and stirred for 2 h at 85 °C then the hot black mixture was poured *carefully* onto ice. The reaction mixture was then extracted with toluene (3 × 20 mL), followed by washing the collected organic layers with sat. $NaHCO_3$ (2 x 15 mL) followed by water (2 x 15 mL). The organic extracts were then dried over anhydrous $MgSO_4$ followed by filtration and the solvent was removed under reduced pressure to give a crude of white solid with crystals on the wall of flask. Pure colourless needle like crystals were obtained by recrystallization from hot methanol to give the target compound (±)-**S1** (0.89 g, 3.90 mmol, 34%). **Mp** 145-149 °C (*Lit.* 147.7-148.8 °C)[55]; **IR** (ν_{max}/cm^{-1}): 2941 (C=C-H, aromatic), 1683 (C=O), 1594 (C=C, aromatic); **^{1}H NMR** (500 MHz, $CDCl_3$): δ 8.19 (dd, *J* = 7.8, 1.3 Hz, 2H), 7.72 (dtd, *J* = 8.9, 7.7, 1.3 Hz, 4H), 7.60 (td, *J* = 7.6, 1.4 Hz, 2H), 4.25 (t, *J* = 3.0 Hz, 2H), 3.23 (t, *J* = 3.0 Hz, 2H) ppm; **^{13}C NMR** (125 MHz, $CDCl_3$) δ 194.5, 140.1, 134.6, 128.9, 128.9, 128.4, 48.9, 32.4 ppm.

Preparation of dinaphthobicyclo-[1,2-*b,f*][3.3.1]-nona-7a,8a-diene-8,16-dione (±)-**5a**

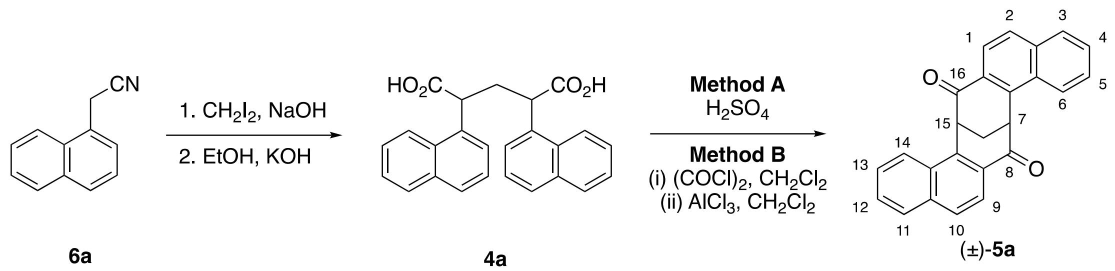

To a mixture of 1-naphthylacetonitrile (5.00 g, 30 mmol) and diiodomethane (1.2 mL g, 15 mmol), was added finely powdered NaOH (1.19 g, 30 mmol). The resulting reaction mixture was then stirred for 2 h at 145 °C, cooled to room temperature, and then a 1:1 mixture of water/ diethyl ether (15 mL) added. The mixture was poured into water (15 mL) and the aqueous layer was extracted with diethyl ether (3 × 10 mL). The combined ethereal layers were dried over anhydrous $MgSO_4$, filtered, and the solvent was removed under reduced pressure to give crude of (±)- and *meso*-2,4-(1-naphthyl)pentanedinitrile as a dark brown oil (55 %, 6.15 g, 16.4 mmol). To a solution of crude dinitrile (6.15 g, 0.016 mol) in ethanol (41 mL), was added a mixture of KOH (11.9 g, 0.21 mol) and water (45 mL). The resulting mixture was stirred at reflux for 20 h, cooled to room temperature, and the ethanol removed under reduced pressure. The aqueous layer was washed with diethyl ether (4 × 20 mL), acidified with concentrated hydrochloric acid, and extracted with ethyl acetate (3 × 20 mL). The organic layers were washed with water (3 × 20 mL), dried over anhydrous $MgSO_4$, filtered and the solvent was then removed uner reduced pressure to give crude (±)- and *meso*-2,4-(1-naphthyl)pentanedioic acids as a brown oily-residue (5.40 g, 92%). This was used crude in the next step (either Method A and B below).

Method A: The crude mixture of (±)-and *meso*-2,4-(1-naphthyl)pentanedioic acids (1.64 g, 3.97 mmol) was dissolved in concentrated sulphuric acid (3.00 mL) and stirred for 2 h at 85 °C and then the hot black mixture was poured *carefully* onto ice. Extraction of this mixture with toluene (3 × 20 mL), followed by washing of the collected organic layers with sat. $NaHCO_3$ (2 x 15 mL) followed by water (2 x 15 mL). The organic layer was then dried over anhydrous $MgSO_4$ followed by filtration and removal of solvent under reduced pressure to give the named product (±)-**5a** (4%).

Method B: To a stirred solution of (±)-and *meso*-2,4-(1-naphthyl)pentanedioic acids (6.00 g, 15.6 mmol) in anhydrous DCM (100 mL) under argon, were added 2.90 eq. of oxalyl chloride (3.90 mL, 45.24 mmol) followed by anhydrous DMF (0.30 mL 4 mmol). After 2 h of stirring at room temperature, the solvent was removed under reduced pressure. Additional DCM was added, and the solvent removed again under reduced pressure (this removed any excess of oxalyl chloride) to give a thick oily brown residue (6.00 g, 14.2 mmol, 91%). Without any further purification, the crude acid chloride (6.00 g, 14.2 mmol) was dissolved in anhydrous DCM (125 mL) and stirring at room temperature. $AlCl_3$ powder (3 equiv., 5.60 g, 42.60 mmol) was then added to the reaction mixture in small portions to prevent the overheating of reaction. The reaction mixture turned from dark brown to dark green upon addition of $AlCl_3$ along with the release of HCl fumes. The reaction mixture was stirred for 3 h at room temperature and then quenched by pouring over ice. Once the ice melted, the solution was extracted with dichloromethane (2 x 50 mL) and the organic layers were combined, dried over $MgSO_4$, filtered and the solvent removed under vacuum to give the title compound (±)-**5a** as green yellowish solid (3.02 g, 8.66 mmol, 61%).

Dinaphthobicyclo-[1,2-*b,f*][3.3.1]-nona-7a,8a-diene-8,16-dione (±)-**5a**

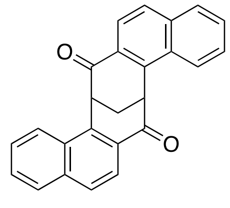

(±)-**5a**

The product (±)-**5a** was recrystallised from hot toluene to obtain white single crystals (Rf [6:4 petroleum ether:ethyl acetate] 0.6). **Mp** 270.2-273.9 °C; **IR** (ν_{max}/cm^{-1}): 2950 (C=C-H, aromatic), 1665 (C=O), 1593 (C=C, aromatic); **^{1}H NMR** (500 MHz, $CDCl_3$): δ 8.75 (d, *J* = 8.6 Hz, 2H), 7.96 (d, *J* = 8.6 Hz, 2H), 7.81 (d, *J* = 8.1 Hz, 2H), 7.76 (d, *J* = 8.6 Hz, 2H), 7.71 (ddd, *J* = 8.4, 6.9, 1.3 Hz, 2H), 7.62 (ddd, *J* = 8.0, 7.0, 1.0 Hz, 2H), 4.93 (t, *J* = 3.0 Hz, 2H), 3.32 (t, *J* = 3.1 Hz, 2H) ppm; **^{13}C NMR** (125 MHz, $CDCl_3$): δ 194.4, 138.9, 136.4, 131.0, 129.00, 128.96, 128.8, 127.8, 126.5, 125.8, 123.3, 43.9, 34.8 ppm; HRMS (ESI) m/z: [M+Na]+ Calcd for $C_{25}H_{16}O_2Na$ 371.1043: Found 371.1042.

Preparation of dinaphthobicyclo-[3,4-*b,f*][3.3.1]-nona-7a,8a-diene-8,16-dione (±)-5b-d.

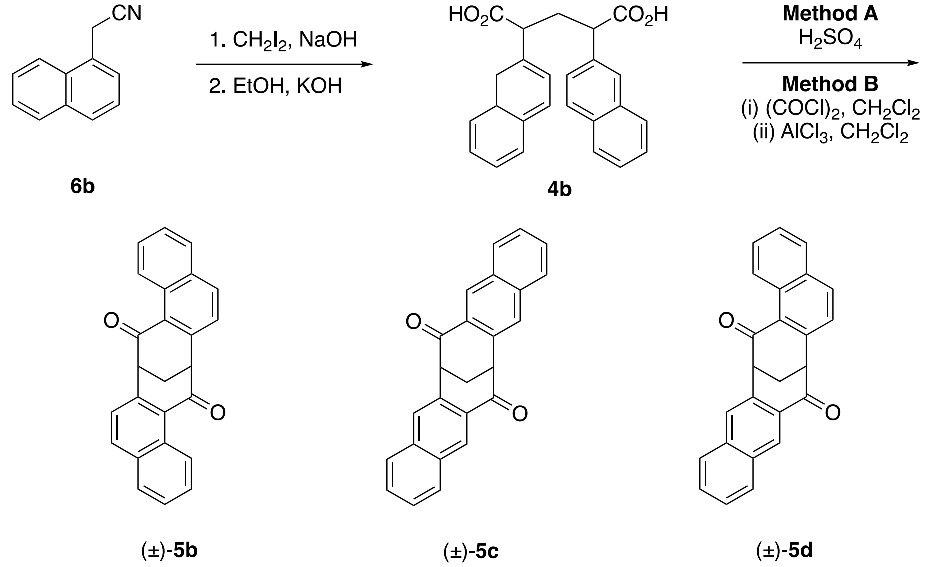

To a mixture of 2-naphthylacetonitrile (5.00 g, 30 mmol) and diiodomethane (1.2 mL g, 15 mmol), was added finely powdered NaOH (1.19 g, 30 mmol). The resulting reaction mixture was stirred for 2 h at 145 °C, cooled to room temperature, and then a 1:1 mixture of water/ diethyl ether (15 mL) added. The mixture was poured into water (15 mL) and the aqueous layer was extracted with diethyl ether (3 × 10 mL). The combined ethereal layers were dried over anhydrous $MgSO_4$, filtered, and the solvent removed under reduced pressure give crude (±)- and *meso*-2,4-(2-naphthyl)pentanedinitrile as a dark brown oil (45 %, 5.06 g, 13.5 mmol). To a solution of crude dinitrile (5.06 g, 13.5 mmol) in ethanol (34 mL), was added a mixture of KOH (9.8 g, 175 mmol) in water (37 mL). The resulting reaction mixture was stirred at reflux for 20 h, cooled to room temperature, and then ethanol was removed under reduced pressure. The aqueous layer was washed with diethyl ether (4 × 20 mL), acidified with concentrated hydrochloric acid and then extracted with ethyl acetate (3 × 20 mL). The organic layers were washed with water (2 × 20 mL), dried over anhydrous $MgSO_4$, filtered and the solvent removed under reduced pressure to give crude (±)- and *meso*-2,4-(2-naphthyl)pentanedioic acids as a brown oily-residue (4.80 g, 86%). This was used crude in the next step (Method A).

Method A: A mixture of **4b** (3.29 g, 7.97 mmol) was dissolved in concentrated sulphuric acid (4.5 mL) and stirred for 2 h at 85 °C, then the hot black mixture was poured onto ice. The reaction mixture with extracted with toluene (3 × 20 mL) and the collected organic layers washed successively with sat. $NaHCO_3$ (2 x 15 mL) and water (2 x 15 mL). The organic extracts were then dried over anhydrous $MgSO_4$, filtered and the solvent removed under reduced pressure to deliver **(±)-5b** as yellowish brown solid with a yield The product **(±)-5b** was then recrystallised from hot toluene to obtain colourless crystals (155 mg, 6%).

Method B: To a stirred solution of **4b** (2.3 g, 5.55 mmol) in anhydrous DCM (70 mL), was added 2.9 eq of oxalyl chloride (1.12 mL, 17.00 mmol) followed by anhydrous DMF (0.3 mL, 4 mmol). After 2 h of stirring at room temperature, the solvent was removed under vacuum and additional DCM was added to remove an excess of oxalyl chloride to give a thick oily brown residue (1.88 g, 4.56 mmol, 83%). Without any further purification, the crude product (1.88 g, 4.56 mmol) was dissolved in anhydrous DCM (125 mL) with stirring at room temperature. $AlCl_3$ powder (1.82 g, 13.68 mmol, 3 equiv.) was added in small portions to prevent the overheating of the reaction mixture. The reaction mixture turned from dark brown to dark green upon addition of $AlCl_3$ along with the release of HCl fumes. The reaction mixture was stirred for 3 h at room temperature and then quenched by pouring over ice. Once the ice melted, the solution was extracted with dichloromethane (2 x 50 mL), the organic layers were combined, dried over $MgSO_4$, filtered and the solvent removed under reduced pressure. This provided a brown solid (0.662 g, 1.9 mmol) which was identified as mixture of (±)-**3b-c.** Extensive chromatography (combiflash) then provided the following isomeric clefts:

Dinaphthobicyclo-[3,4-*b,f*][3.3.1]-nona-7a,8a-diene-8,16-dione (±)-**5b**[2]

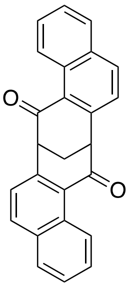

(±)-**5b**

(±)-**5b** was obtained as a white solid (0.089 g, 0.255 mmol, 6%) (Rf [7:3 petroleum ether:ethyl acetate] 0.5). **Mp** 176.9-178.4 °C decomp (Lit. **Mp** for **(-)-5b** 212-214 °C)[2]; **IR** (ν_{max}/cm^{-1}): 2922 (C=C-H, aromatic), 1664 (C=O), 1591 (C=C, aromatic). **^{1}H NMR** (500 MHz, $CDCl_3$): δ 9.40 (d, *J* = 8.8 Hz, 2H), 7.95 (d, *J* = 8.4 Hz, 2H), 7.74 (d, *J* = 8.0 Hz, 2H), 7.72 – 7.49 (m, 4H), 7.51 – 7.37 (m, 2H), 4.25 (t, *J* = 3.0 Hz, 2H), 3.22 (t, *J* = 3.0 Hz, 2H) ppm. **^{13}C NMR** (125 MHz, $CDCl_3$): δ 196.8, 143.2, 135.8, 134.1, 132.0, 129.5, 128.6, 127.1,

126.49, 126.44, 122.3, 53.5, 33.8 ppm; HRMS (ESI) m/z: [M+Na]+ Calcd for $C_{25}H_{16}O_2Na$ 371.1043: Found 371.1042.

Dinaphthobicyclo-[2,3-*b,f*][3.3.1]-nona-7a,8a-diene-8,16-dione (±)-**5c**

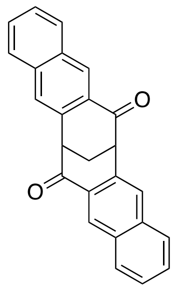

(±)-**5c**

(±)-5c was recrystalised via slow evaporation from dichloromethane and hexane to give transparent crystals (0.034 g, 0.1 mmol, 2%) (Rf [7:3 petroleum ether:ethyl acetate] 0.5). **Mp** 280.4-281.3 °C decomp; **IR** (ν_{max}/cm^{-1}): 2930 (C=C-H, aromatic), 1680 (C=O), 1590 (C=C, aromatic); **^{1}H NMR** (400 MHz, $CDCl_3$): δ 8.55 (s, 1H), 7.96 (s, 1H), 7.84 (ddd, J = 12.3, 8.3, 0.6 Hz, 2H), 7.55 (ddd, *J* = 8.2, 6.8, 1.2 Hz, 1H), 7.44 (ddd, *J* = 8.1, 6.9, 1.2 Hz, 1H), 4.30 (t, *J* = 2.9 Hz, 1H), 3.09 (t, *J* = 3.0 Hz, 1H) ppm; **^{13}C NMR** (100 MHz, $CDCl_3$): δ 195.2, 136.3, 134.6, 132.7, 130.5, 129.9, 129.2, 127.9, 127.8, 127.3, 126.9, 49.2, 31.6 ppm; HRMS (ESI) m/z: [M+Na]+ Calcd for $C_{25}H_{16}O_2Na$ 371.1043: Found 371.1042.

Naphtho-[3,4-*b*]-naphtho-[2,3-*f*]-bicyclo- [3.3.1]-nona-7a,8a-diene-8,16-dione (±)-**5d**

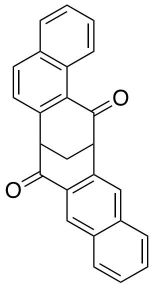

(±)-**5d**

(±)-3d was recrystalised from ethyl acetate and hexane to give white crystals (0.393 g, 1.1 mmol, 25%) (Rf [7:3 petroleum ether:ethyl acetate] 0.5). **Mp** 192.7-195.4 °C; **IR (ν_{max}/cm^{-1}):** 2928 (C=C-H, aromatic), 1662 (C=O), 1589 (C=C, aromatic); **^{1}H NMR** (400 MHz, $CDCl_3$): δ 9.43 (d, *J* = 8.8 Hz, 1H), 8.52 (s, 1H), 7.95 (d, *J* = 11.0 Hz, 2H), 7.81 (dd, *J* = 11.6, 8.4 Hz, 2H), 7.73 (d, *J* = 8.0 Hz, 1H), 7.64 – 7.48 (m, 3H), 7.42 (dd, J = 15.6, 7.7 Hz, 2H), 4.25 (d, *J* = 8.1 Hz, 2H), 3.20 (dt, *J* = 13.2, 3.0 Hz, 1H), 3.05 (dt, *J* = 13.3, 2.7 Hz, 1H) ppm; **^{13}C NMR** (100 MHz, $CDCl_3$): δ 197.3, 194.6, 142.3, 136.2, 135.9, 135.2, 134.0, 132.7, 132.0, 130.5, 129.9, 129.5, 129.2, 128.6, 127.7, 127.57, 127.1, 126.9, 126.8, 126.6, 126.5, 123.4, 51.3, 32.5 ppm; HRMS (ESI) m/z: [M+Na]+ Calcd for $C_{25}H_{16}O_2Na$ 371.1043: Found 371.1042.

Synthesis of (7*S*,8*R*,15*S*,16*R*)-7,8,15,16-tetrahydro-8,16-methanocycloocta[1,2-a:5,6-a'] dinaphthalene-7,15-diol (±)-7a.

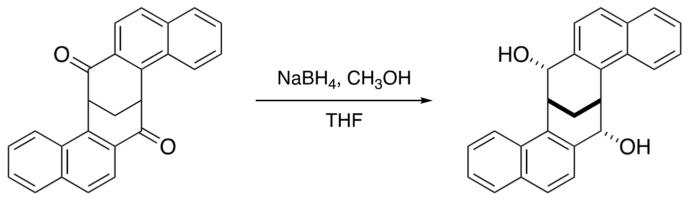

(±)-**5a** (±)-**7a**

A solution of (±)-**5a** (100 mg, 0.290 mmol, 1 eq.) and sodium borohydride (53.7 mg, 1.4 mmol, 4.9 eq), in THF (2.5 mL) and methanol (2.5 mL) was stirred at room temperature for 48 h. After this time, the solvent was removed under reduced pressure and ethyl acetate (50 mL) and water (40 mL) added. The organic layer was washed with water (3 x 20 mL), dried over $MgSO_4$, filtered and the solvent removed under vacuum to give **(±)-7a** as a white solid and recrystallized from hot methanol (52 mg, 51%) (Rf [6:4 petroleum ether:ethyl acetate] 0.3). **Mp** 260.8 - 261.5 °C decomp; **IR** (ν_{max}/cm^{-1}): 3373 (O-H), 2915 (C=C-H, aromatic), 1506 (C=C, aromatic); ^{1}H NMR (500 MHz, $CDCl_3$): δ 8.49 (d, *J* = 8.6 Hz, 2H), 7.77 (d, *J* = 7.9 Hz, 2H), 7.70 (q, *J* = 8.6 Hz, 4H), 7.57 (ddd, *J* = 8.5, 6.9, 1.4 Hz, 2H), 7.50 – 7.43 (m, 2H), 5.48 (dd, *J* = 11.9, 6.1 Hz, 2H), 4.37 (dt, *J* = 6.3, 3.2 Hz, 2H), 2.62 (t, *J* = 3.3 Hz, 2H), 1.27 (d, *J* = 12.1 Hz, 2H) ppm; **^{13}C NMR** (125MHz, $CDCl_3$): δ 137.2, 133.2, 132.9, 130.2, 128.4, 128.2, 126.1, 125.8, 125.2, 75.1, 34.1, 30.5 ppm; HRMS (ESI) m/z: [M+Na]+ Calcd for $C_{25}H_{20}O_2Na$ 375.1361: Found 375.1355.

Synthesis of (7*R*,8*S*,15*R*,16*S*)-7,8,15,16-tetrahydro-7,15-methanocycloocta[1,2-a:5,6-a']dinaphthalene-8,16-diol (±)-7b.

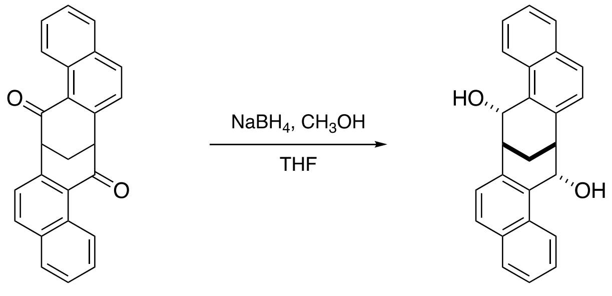

(±)-**5b** (±)-**7b**

A solution of (±)-**5b** (84 mg, 0.24 mmol, 1 eq) and sodium borohydride (45.4 mg, 1.4 mmol, 4.9 eq), in THF (2.0 mL) and methanol (2.0 mL) was stirred at room temperature for 48 h. After this time, the solvent was removed under reduced pressure and ethyl acetate (50 mL) and

water (40 mL) were added. The organic layer was washed with water (3 x 20 mL), dried over $MgSO_4$, filtered and the solvent removed under vacuum to give **(±)-7b** as a white solid (40 mg, 47%). **Mp** 280.4 - 281.3 °C (Lit **Mp** for **(-)-7b-** 227 - 229 °C)[2]; **IR** (ν_{max}/cm^{-1}): 3427 (O-H), 2929 (C=C-H, aromatic), 1560 (C=C, aromatic); **^{1}H NMR** (500 MHz, $CDCl_3$): δ 8.65 (d, *J* = 8.6 Hz, 2H), 7.78 (t, *J* = 15.7 Hz, 2H), 7.70 (d, *J* = 8.5 Hz, 4H), 7.49 (dd, *J* = 12.8, 4.9 Hz, 2H), 7.46 – 7.37 (m, 2H), 5.81 (dd, *J* = 11.5, 7.1 Hz, 2H), 3.75 – 3.73 (m, 2H), 2.40 (t, *J* = 3.1 Hz, 2H), 1.60 (d, *J* = 11.6 Hz, 2H) ppm; **^{13}C{1H} NMR** (125 MHz, $CDCl_3$) δ 136.6, 133.7, 133.4, 132.6, 131.7, 128.2, 127.3, 127.2, 126.0, 125.1, 70.4, 41.4, 28.8 ppm; HRMS (ESI) m/z: [M+Na]+ Calcd for $C_{25}H_{20}O_2Na$ 375.1361: Found 375.1355.

1H NMR (500 MHz, $CDCl_3$) for (±)-**S1**

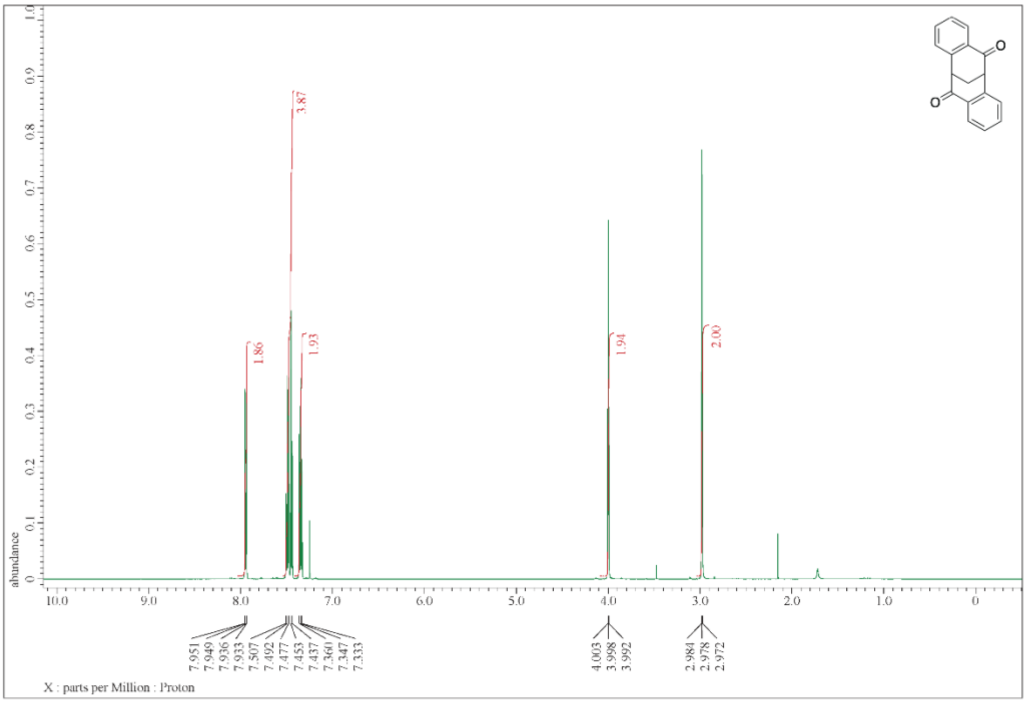

13C{1H} NMR (125 MHz, $CDCl_3$) for **(±)-S1**

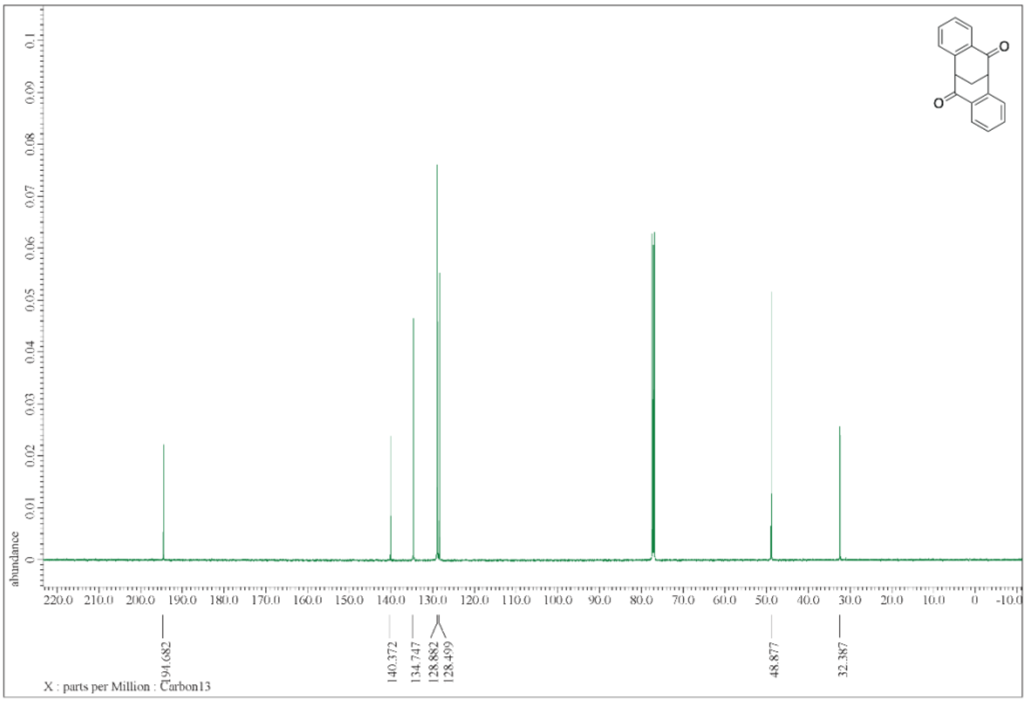

1H NMR (500 MHz, $CDCl_3$) for **(±)-5a**

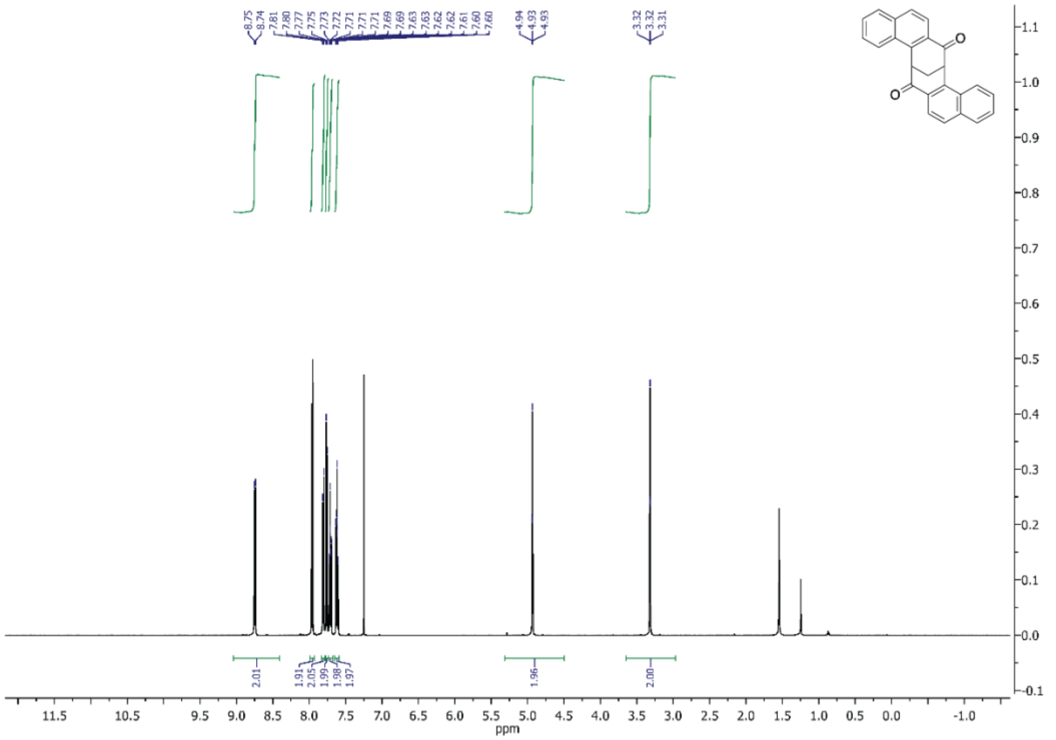

13C{1H} NMR (125 MHz, $CDCl_3$) for **(±)-5a**

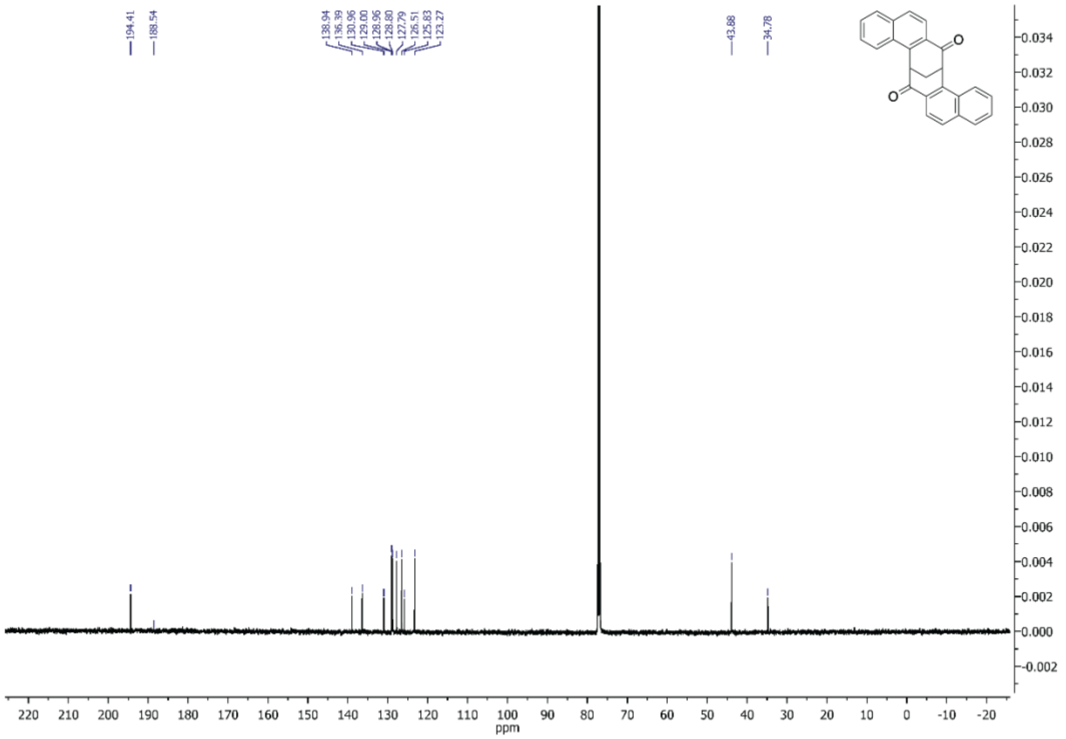

1H NMR (500 MHz, $CDCl_3$) for **(±)-5b**

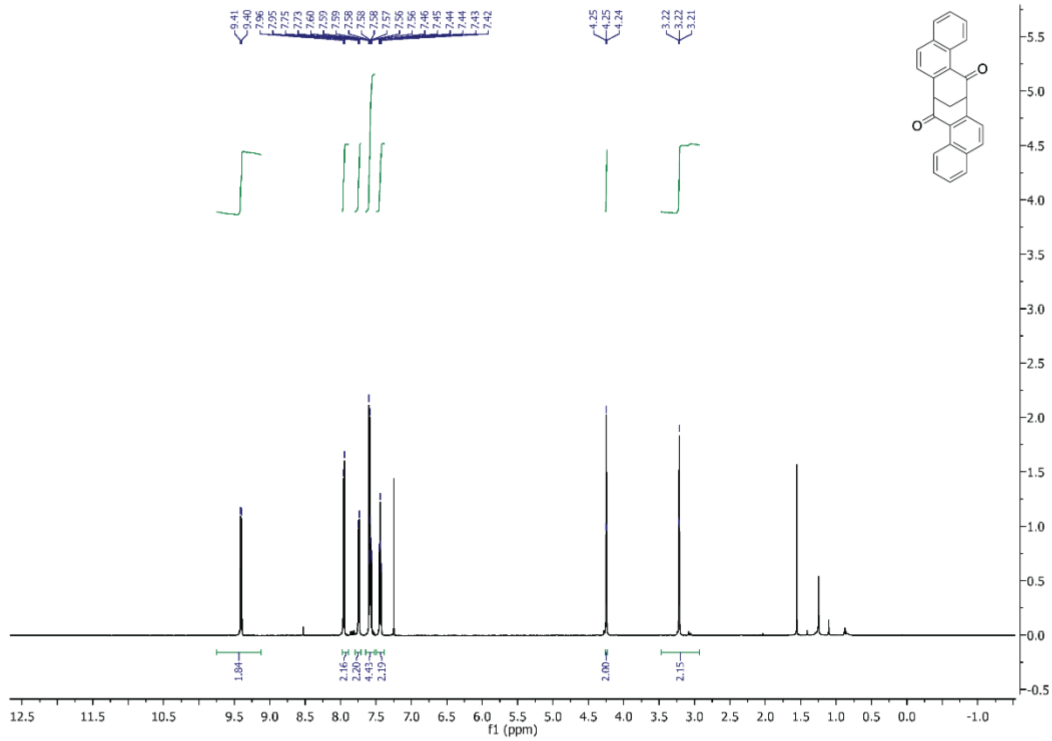

13C{1H} NMR (125 MHz, $CDCl_3$) for **(±)-5b**

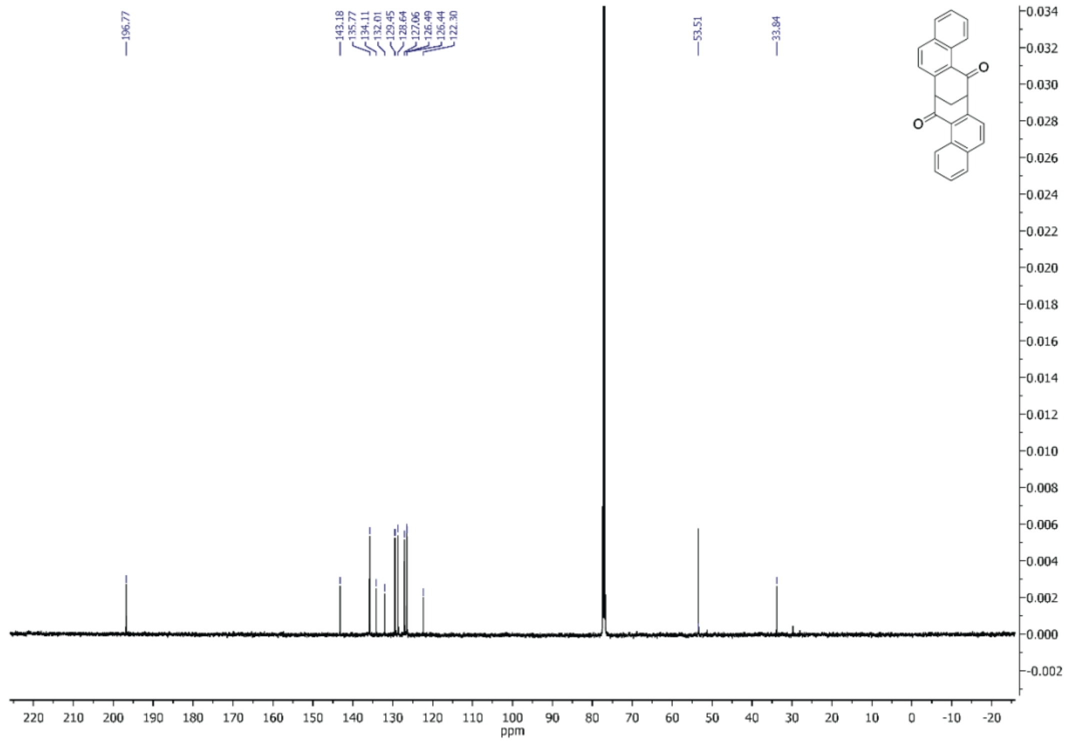

1H NMR (400 MHz, CDCl$_3$) for **(±)-5c**

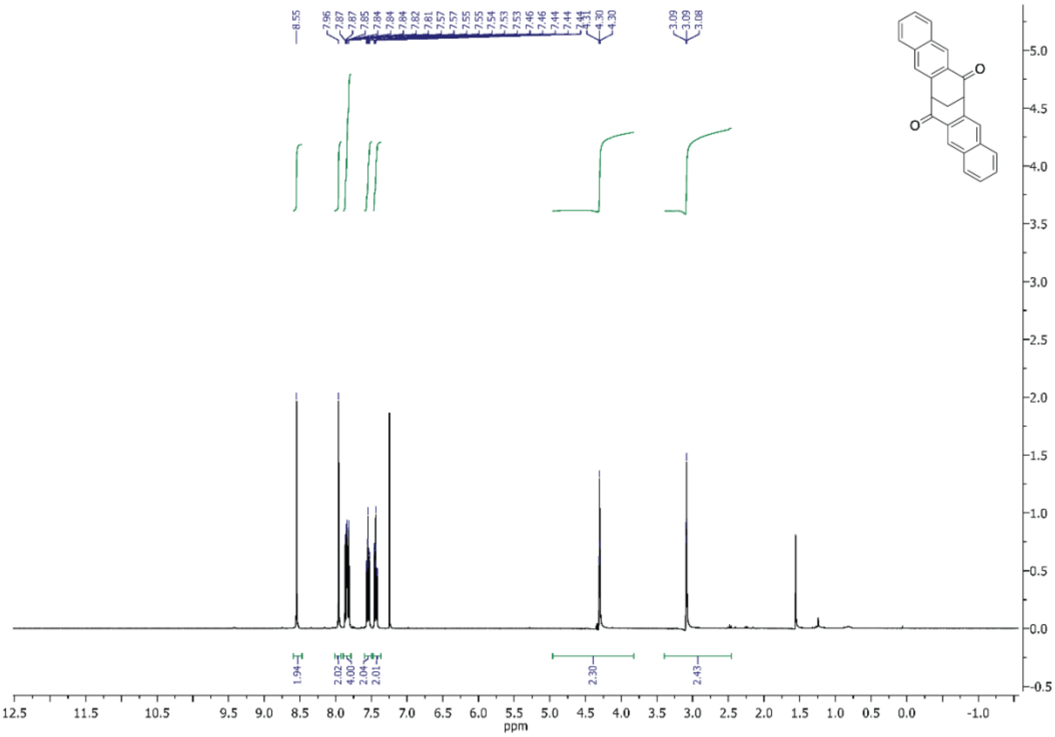

13C{1H} NMR (100 MHz, CDCl$_3$) for **(±)-5c**

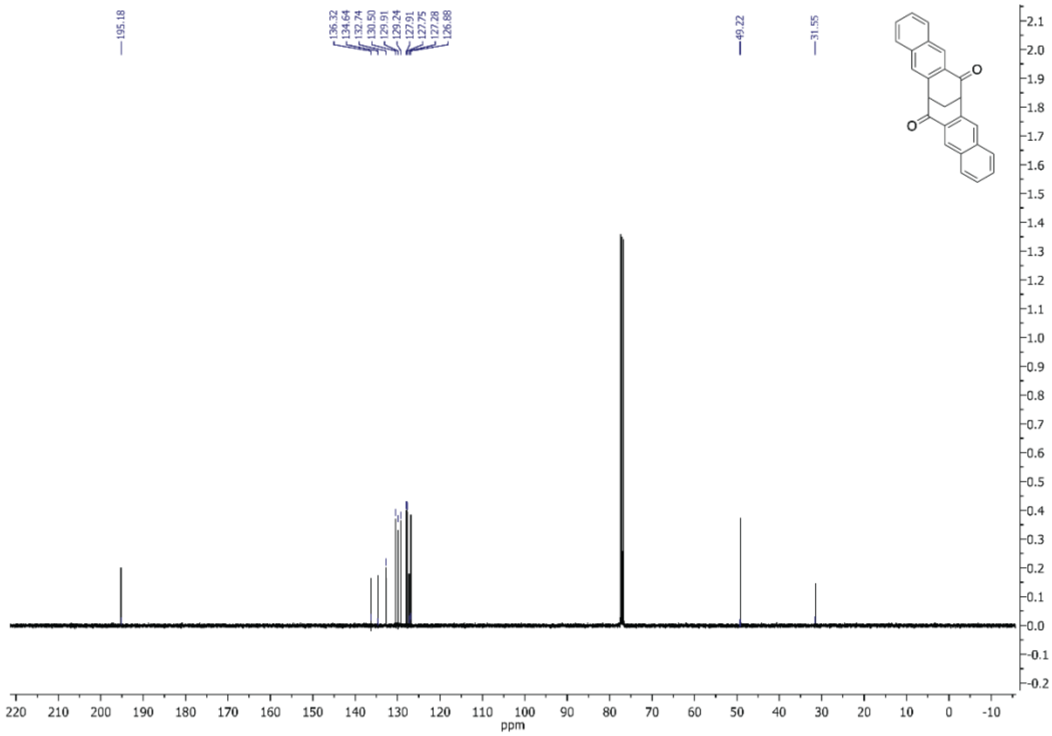

1H NMR (400 MHz, $CDCl_3$) for **(±)-5d**

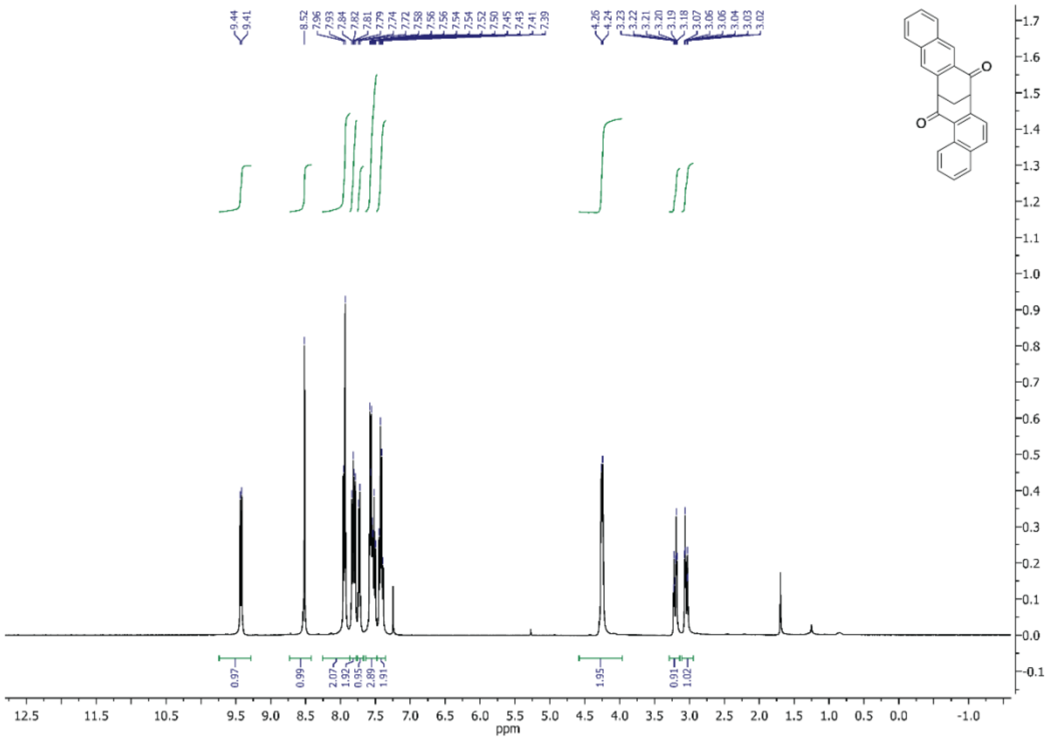

13C{1H} NMR (100 MHz, $CDCl_3$) for **(±)-5d**

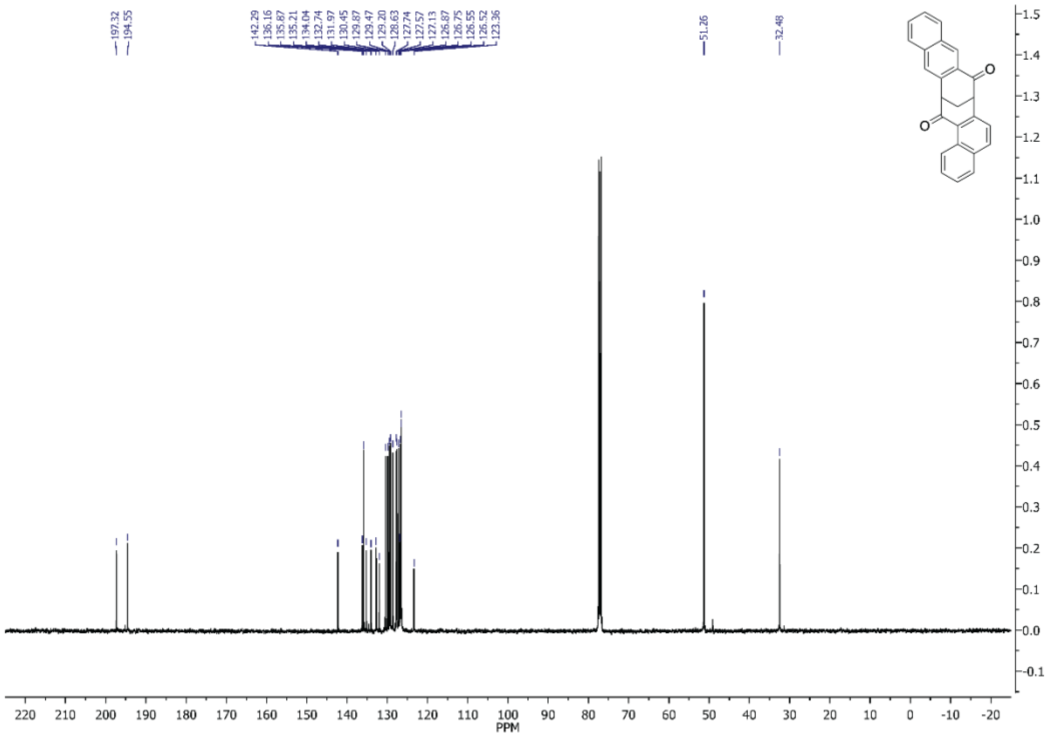

1H NMR (500 MHz, $CDCl_3$) for **(±)-7a**

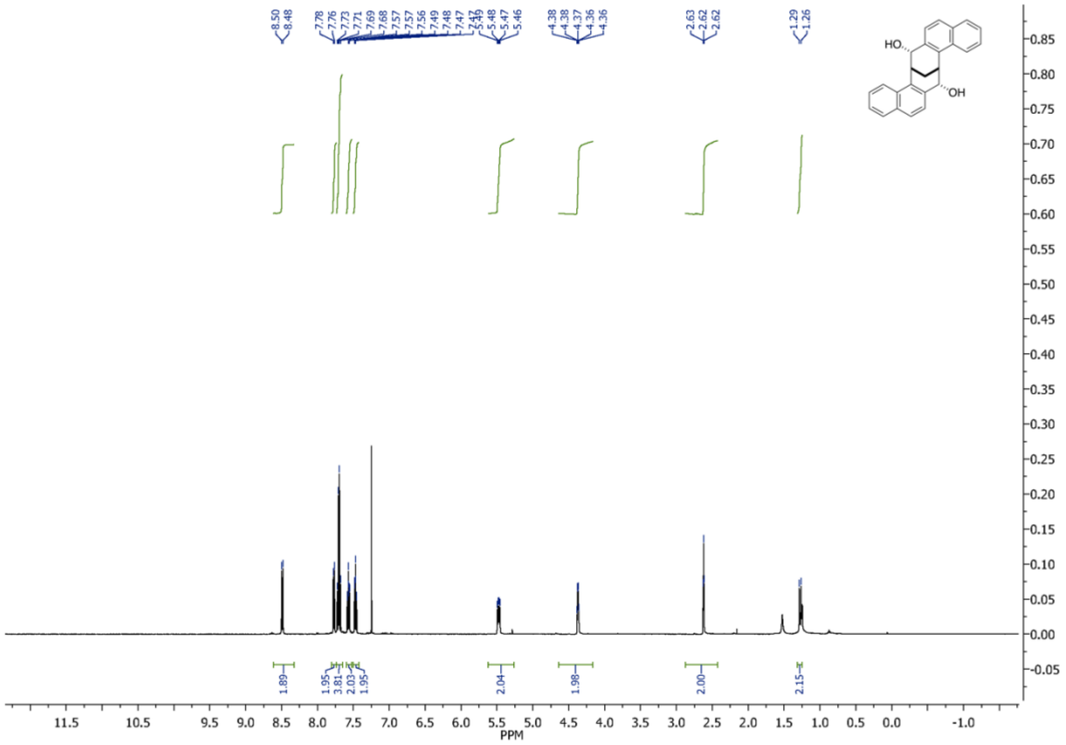

13C{1H} NMR (125 MHz, $CDCl_3$) for **(±)-7a**

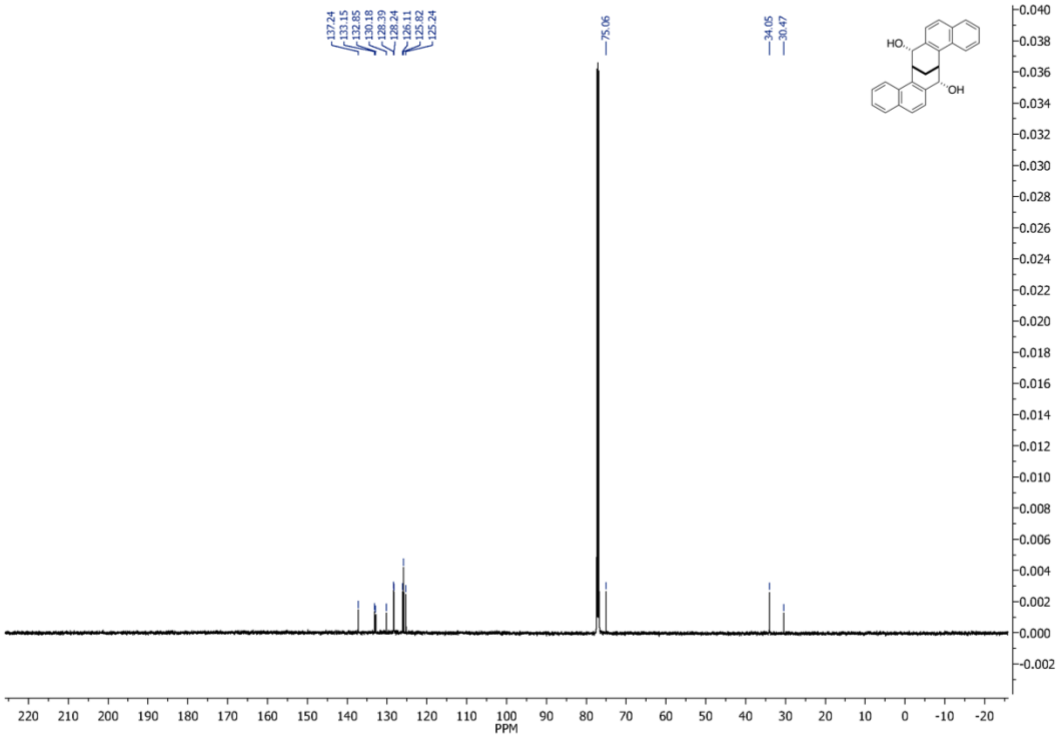

1H NMR (500 MHz, $CDCl_3$) for **(±)-7b**

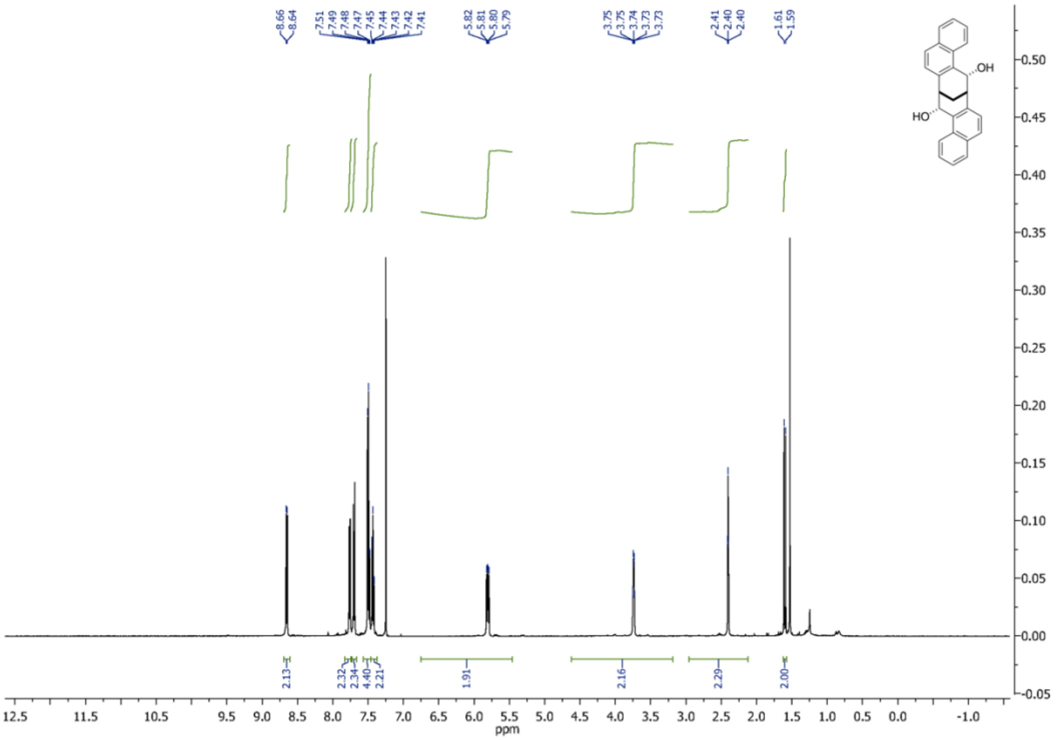

13C{1H} NMR (125 MHz, $CDCl_3$) for **(±)-7b**

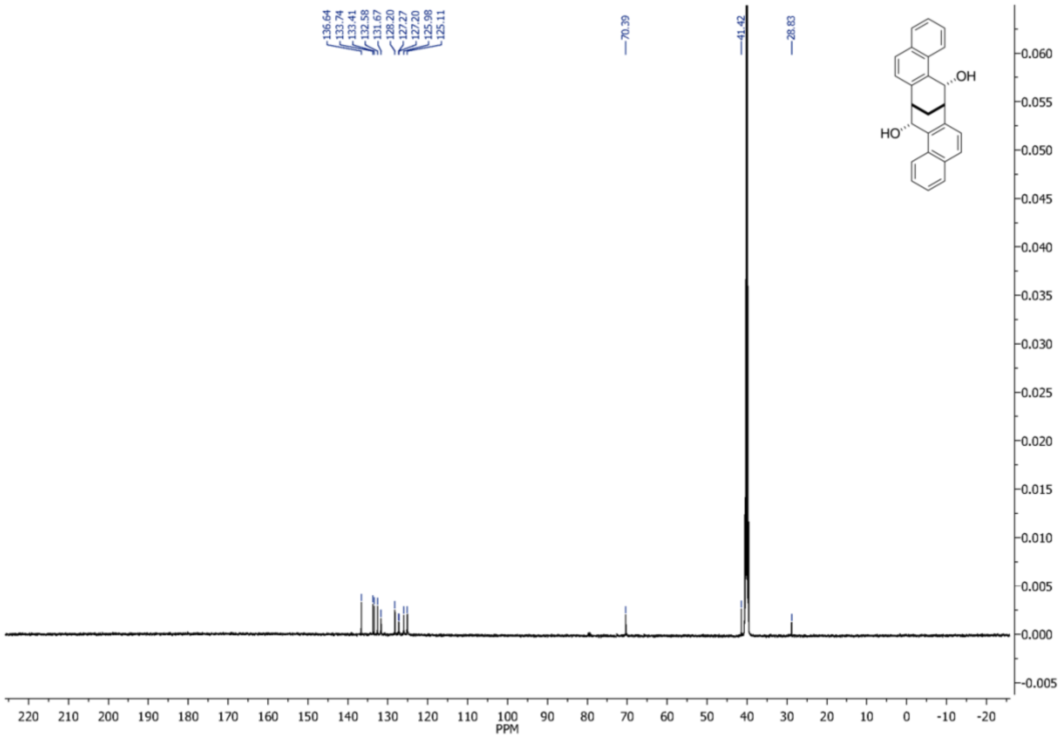

X-ray Crystallography

Diffraction data were collected on a Bruker Apex 2 CCD diffractometer[3] at 150 K using Mo-Kα radiation ((±)-***5a***, (±)-***5b*** & (±)-***7a***) or a Rigaku 007HF AFC11 diffractometer[4] at 100 K using Cu-Kα radiation ((±)-***5c*** & (±)-***5d***). The data were corrected for absorption and Lp effects via a multi-scan method based on repeated data.[3,4] The structures were solved by a dual space charge flipping algorithm[5] and refined by full matrix-least-squares procedures.[6] Full data collection and crystal data details are presented in Tables S1–S5 below and the following paragraphs for the individual structures. For each structure additional views of the molecule and the packing are provided in the figures below. CCDC 2179076, 2179077, 2183730, 2183731, & 2212745 contain the supplementary crystallographic data for this paper. These data can be obtained free of charge from The Cambridge Crystallographic Data Centre via www.ccdc.cam.ac.uk/structures.

For (±)-***5a***, the data are of good quality and the refinement proceeded smoothly. Hence a good *R*-factor and other quality indicators. The absolute structure could not be reliably determined due to the lack of any heavy atoms with this radiation type.

For (±)-***5b***, however, the data were of much poorer quality, with underlying and unresolved twinning effects. One reason for this is likely to be the fact that all three unit cell axes are about the same length in the tetragonal crystal system. In addition, due to the space group symmetry, the molecule lies over two symmetry elements, so only a quarter is unique, and this leads to inherent disorder with overlapping molecules. Hence atoms H(1A)/H(1B)/H(6)/C(7)/H(7)/C(8)/H(8)/O(1) needed to be refined at half weight. Geometrical and *U* value restraints were applied in this case. Overall the *R*-factor and other quality indicators were marginal, but the molecular connectivity has been established.

For (±)-***5c*** and (±)-***7a***, also symmetrical molecules, again the data are of high quality and the structures were refined routinely.

For (±)-***5d*** which is an example of an unsymmetrical molecule, the data were again poor. Here, the was some ambiguity regarding the crystal system. The unit cell is metrically orthorhombic, and the structure was progressed in that crystal system, with Platon's symmetry analysis also suggesting this is most likely correct. Restraints were applied to all vibration parameters and to some of the geometry. An equally poor outcome could be obtained in monoclinic symmetry. The final quality indicators are very poor in this case. It

seems there is underlying twinning and/or disorder in the aromatic rings which could not be resloved. As such this structure should be regarded as provisional. The supporting data from other characterisation techniques do fully support the proposed structure.

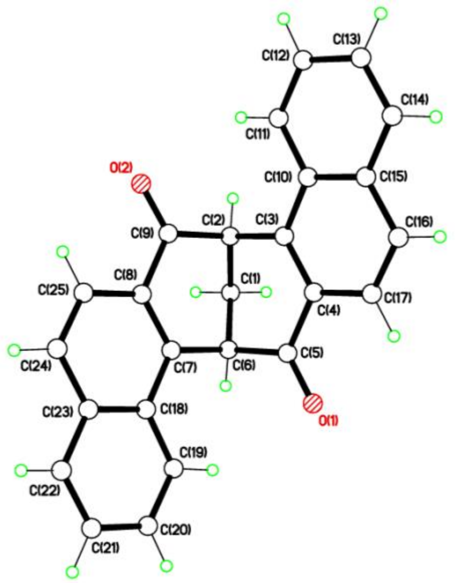

Figure S1. Alternative view of (±)-***5a***.

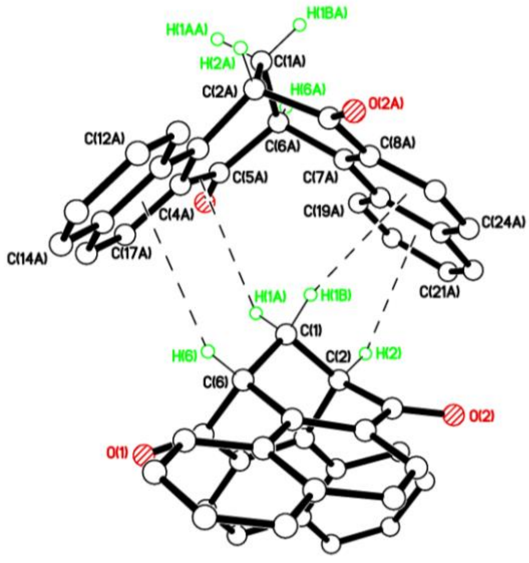

Figure S2. Molecules of (±)-***5a*** stack with C–H···π interactions between the CH groups on the cleft bridge and the underside of the aromatic rings in the next molecule: H(1A)···centroid of atoms C(4) and C(5) = 2.83 Å; H(1B)···centroid of ring C(7)/C(8)/C(18)/C(23) > C(25) = 2.79 Å; H2···centroid of ring C(18) > C(23) = 2.87Å; H(6)···centroid of ring (C3)/C(4)/C(10)/C(15) > C(17) = 3.01 Å

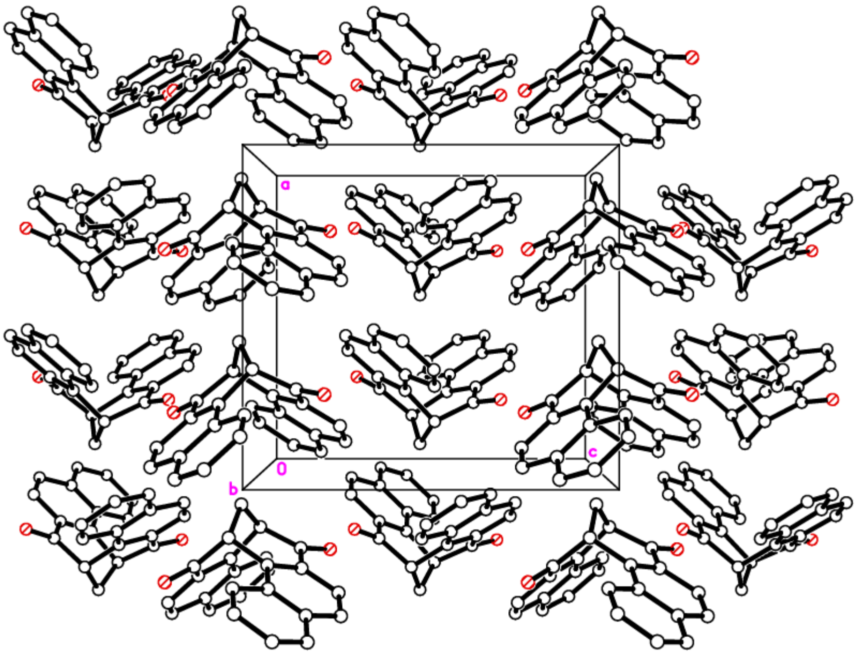

Figure S3. Packing plot for (±)-***5a***. viewed parallel to *b* showing molecules stacking parallel to the *a* axis.

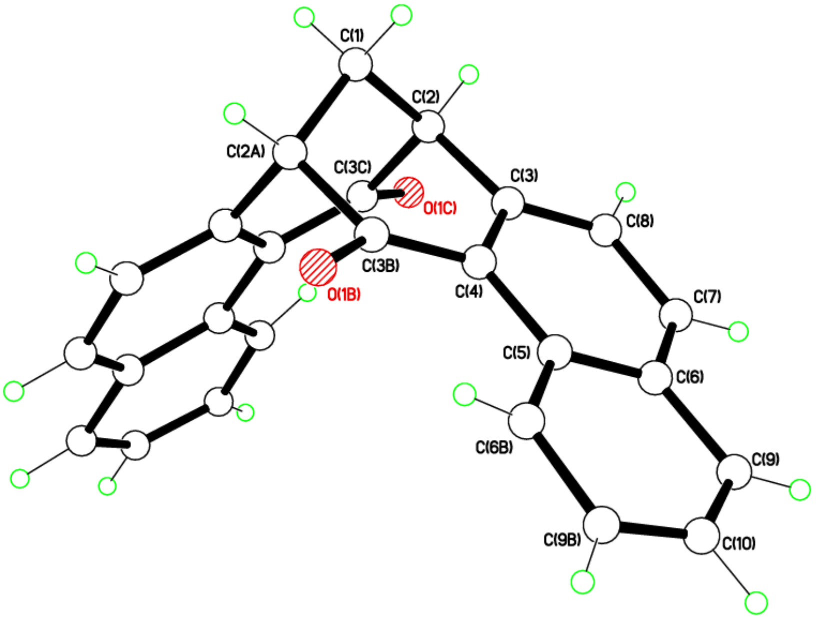

Figure S4. View of (±)-***5b***.

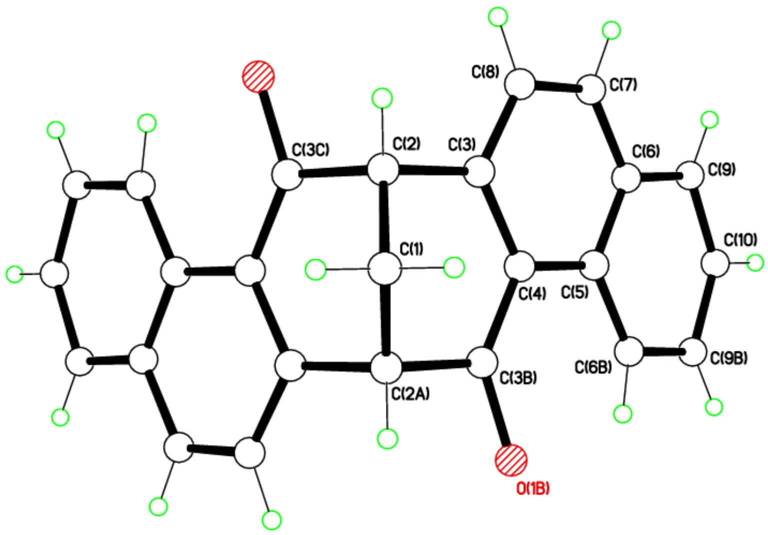

Figure S5. Alternative view of (±)-***5b***.

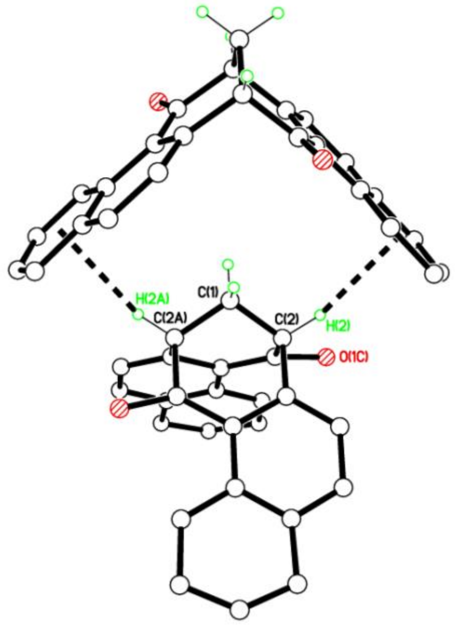

Figure S6. For (±)-***5b*** C(2)–H(2)···π interactions between the CH groups on the cleft bridge and the underside of the terminal aromatic rings in the next molecule at 2.61 Å for the H···centroid distance. Note H atoms on C(1) are not involved.

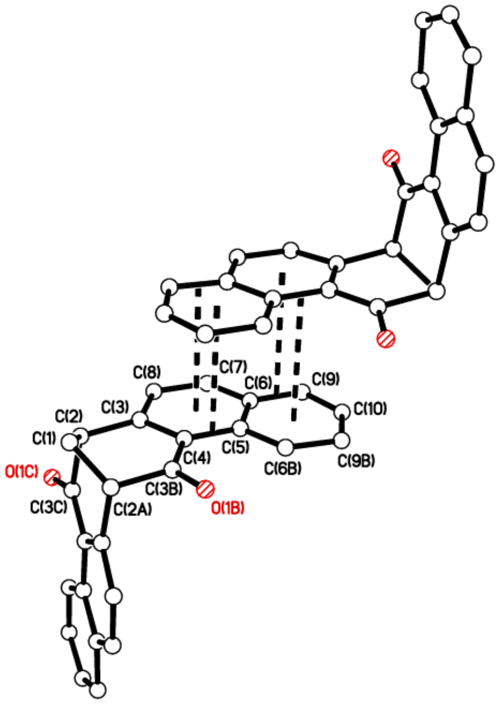

Figure S7. For (±)-***5b***, π···π interactions between naphthyl rings with ring centroid to bond centroid distances of approx. 3.46 Å.

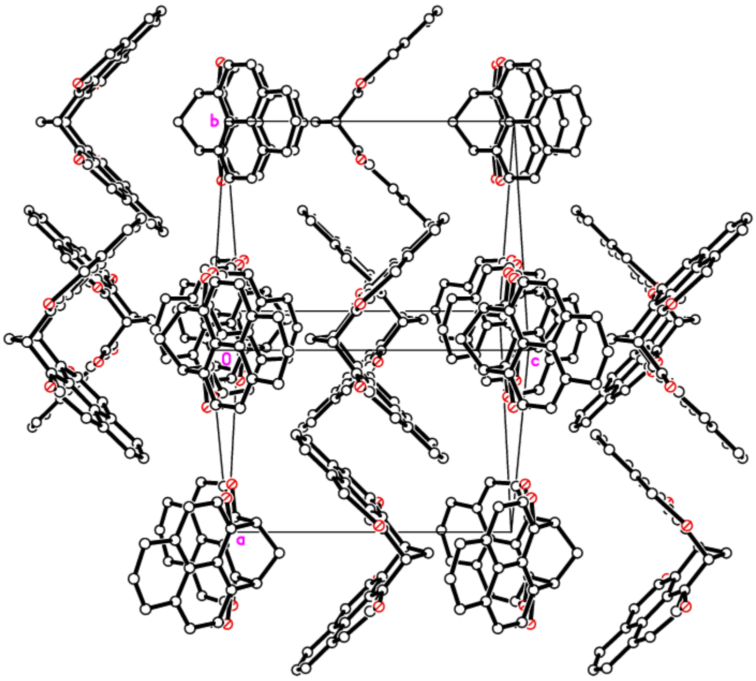

Figure S8. For (±)-***3b*** molecules stack in columns along the *c* axis and molecules along this column are rotated by 90°. The π···π interactions, shown in Figure 5 above, occur between columns.

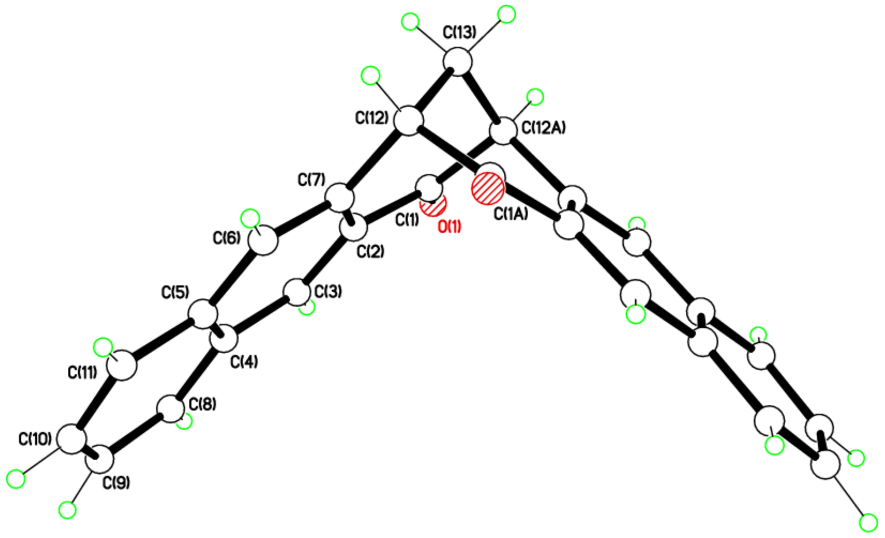

Figure S9. View of (±)-***5c***.

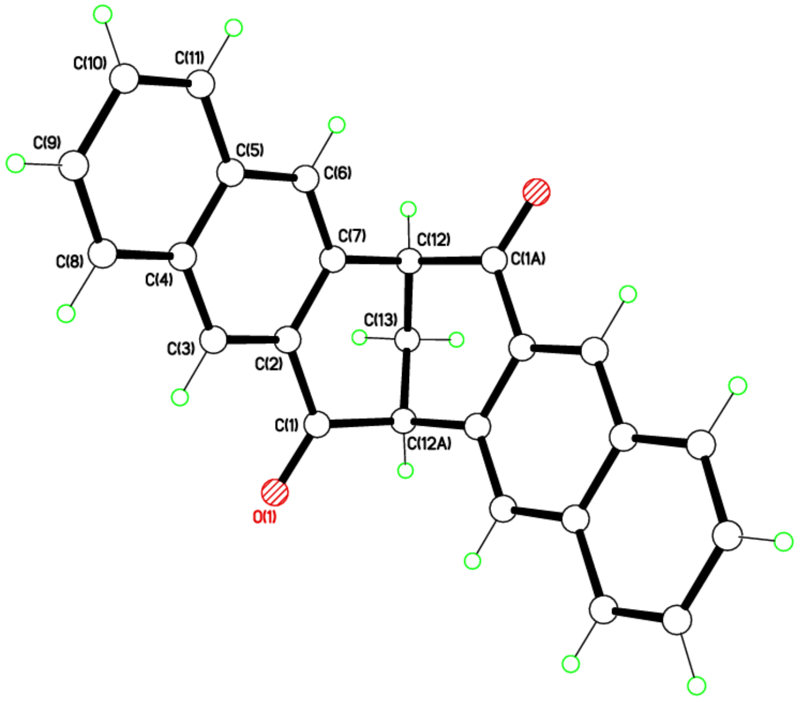

Figure S10. Alternative view of (±)-***5c***.

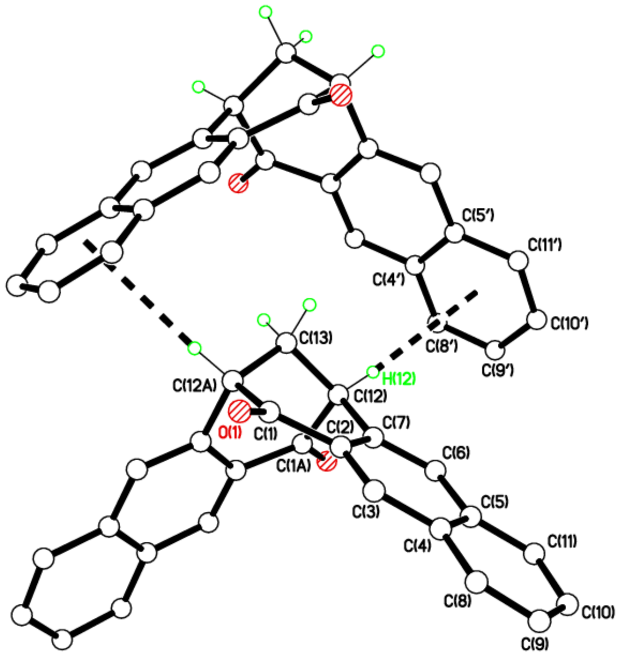

Figure S11. For (±)-***5c*** C(12)–H(12)···π interactions between the CH groups on the cleft bridge and the underside of the terminal aromatic rings in the next molecule at 2.86 Å for the H···centroid distance. Note H atoms on C(13) are not involved.

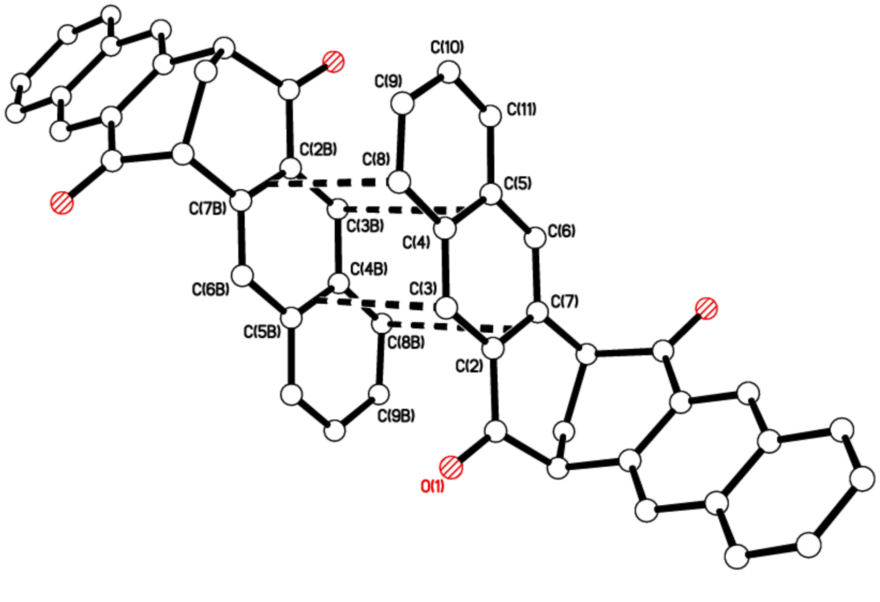

Figure S12. For (±)-***5c***, π···π interactions with distances between centroids of C(2)/C(7) and C(4)/C(5) to atoms C(8B) and C(3B) of 3.480 and 3.388 Å, respectively. The molecules are related by inversion.

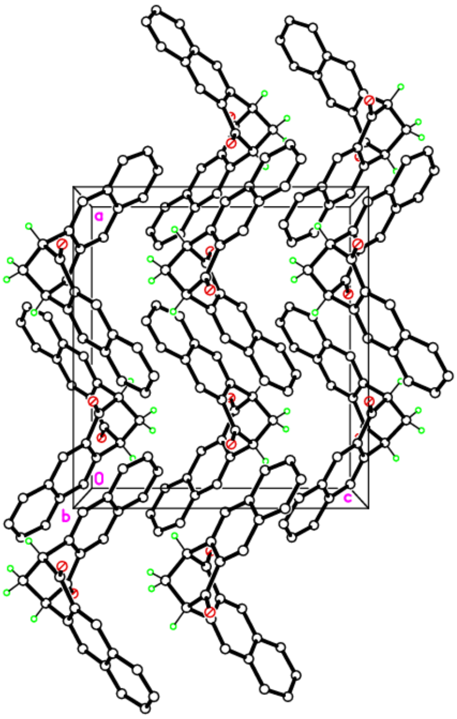

Figure S13. Packing plot of (±)-***5c*** showing π···π stacking in the *c* direction with neighbouring molecules rotated by 90°.

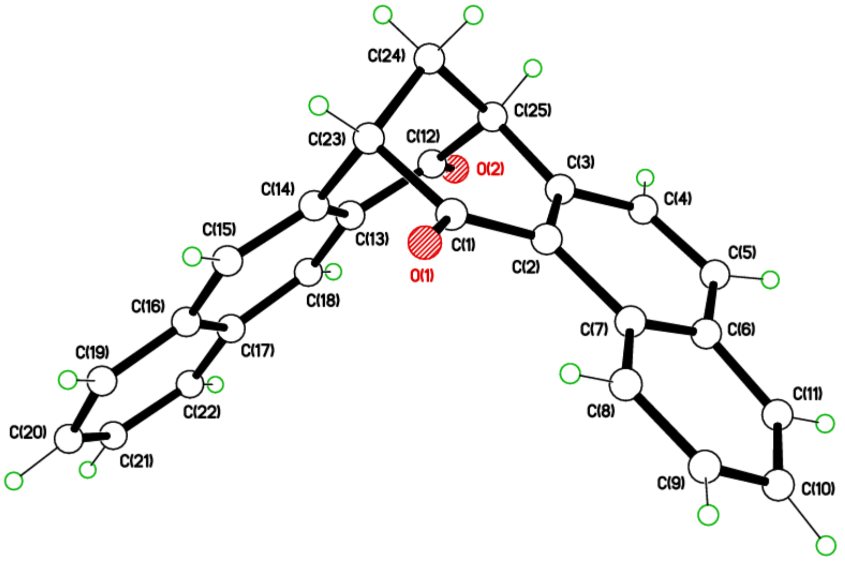

Figure S14. View of (±)-***5d****.*

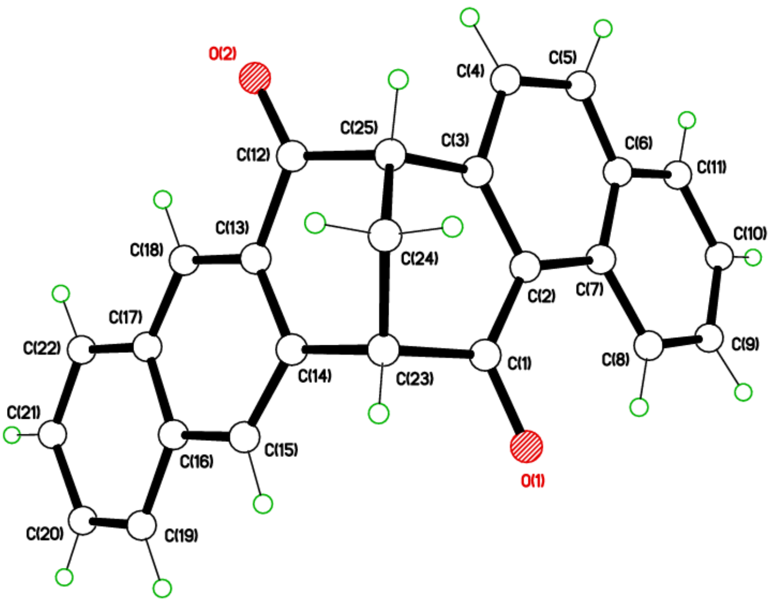

Figure S15. Alternative view of (±)-***5d***.

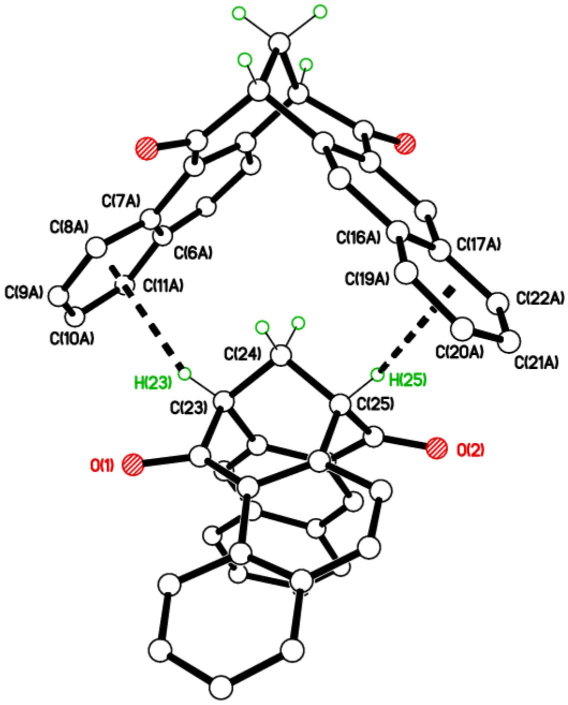

Figure S16. For (±)-***5d*** C(23/25)–H(23/25)···π interactions between the CH groups on the cleft bridge and the underside of the terminal aromatic rings in the next molecule at 2.72 & 2.92 Å respectively for the H···centroid distance. Note H atoms on C(24) are not involved.

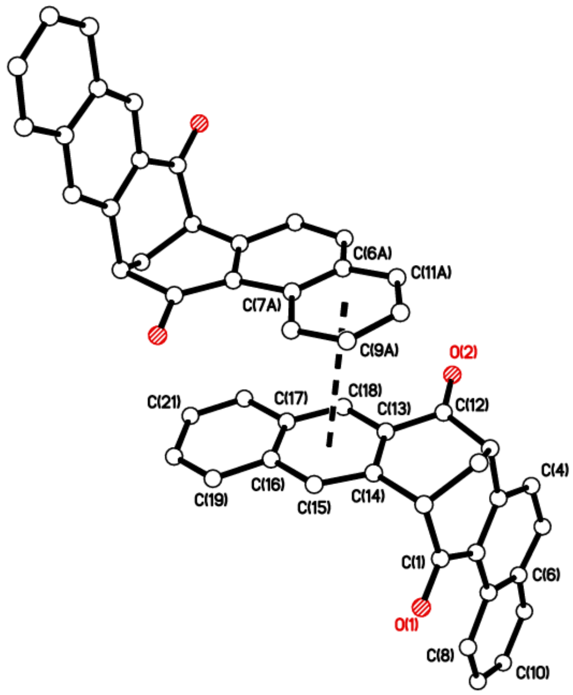

Figure S17. For (±)-***5d***, π···π interactions with distances between ring centroids of *ca.* 3.62 Å, and closest C···C contact of *ca.* 3.50 Å.

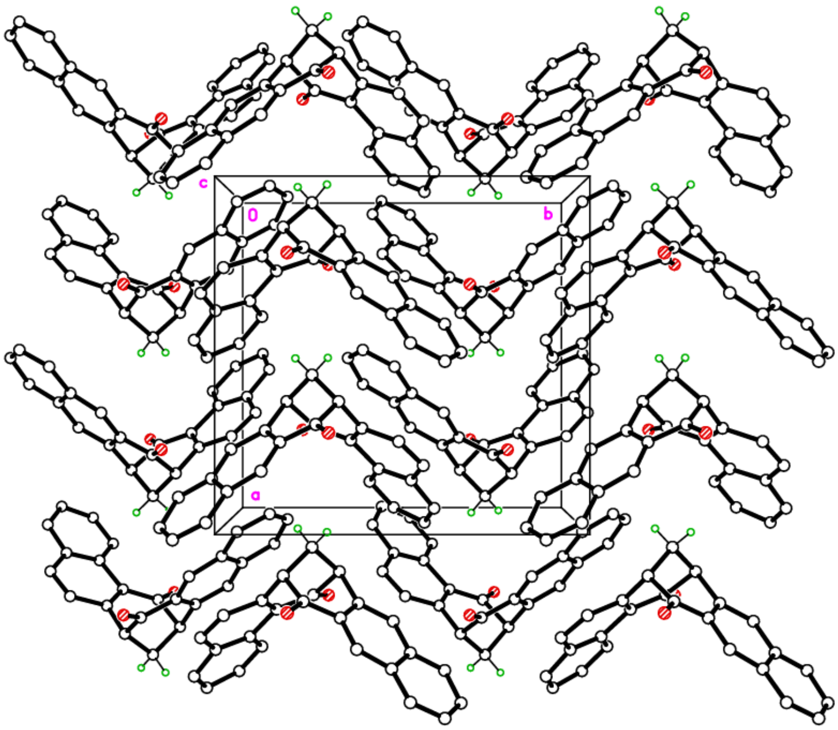

Figure S18. Packing plot of (±)-***5d*** showing π···π stacking in the *b* direction and with stacking in the *a* direction with neighbouring molecules rotated by 90°.

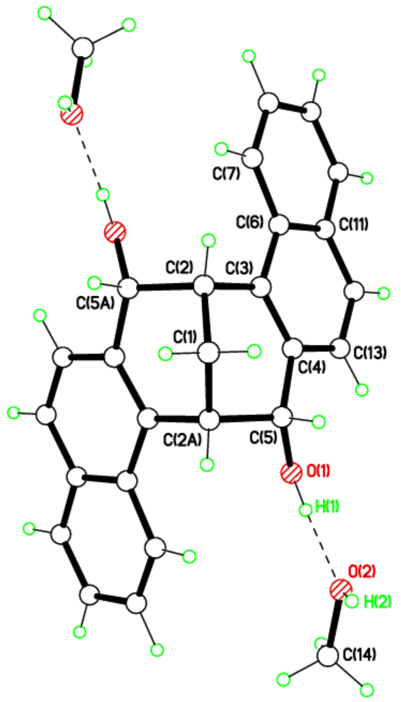

Figure S18. The crystal structure of (±)-**7*a***. Half the cleft molecule and one methanol of crystallisation comprise the asymmetric unit.

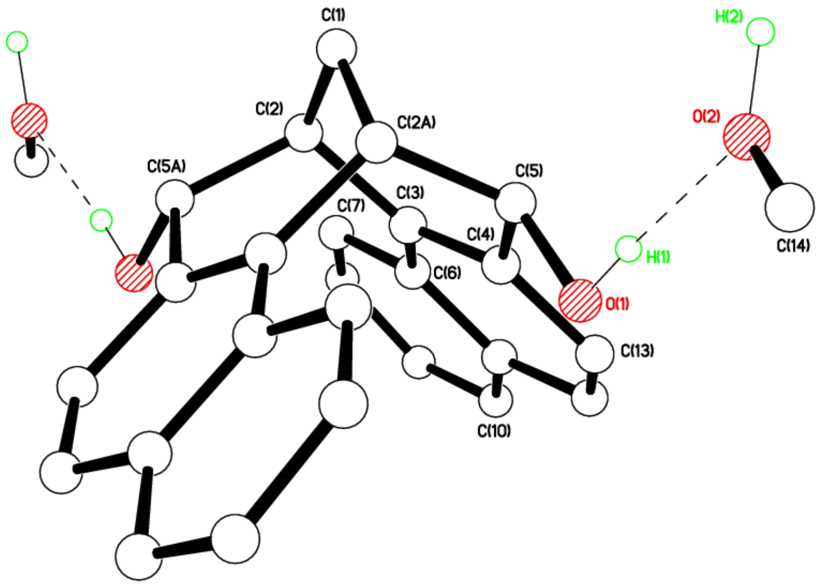

Figure S19. Alternative view of the crystal structure of (±)-**7*a***.

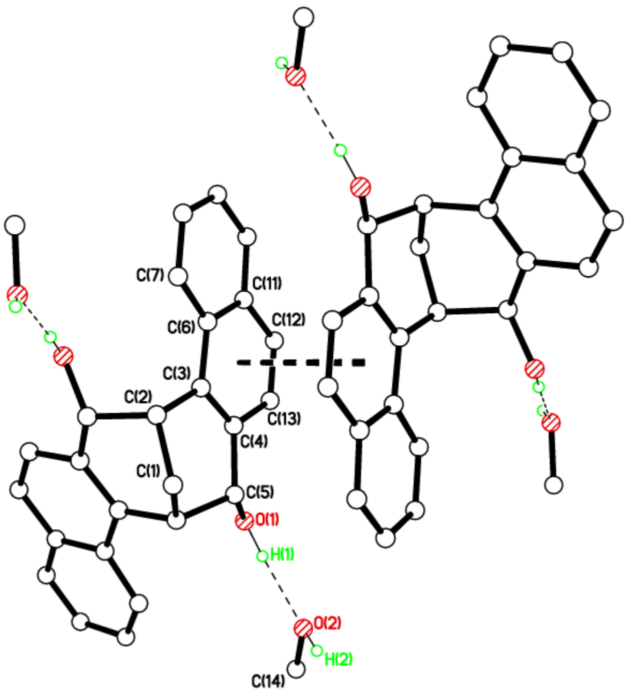

Figure S20. π···π interactions between neighbouring pairs of the molecules in the crystal structure of (±)-**7*a***. The centroid to centroid separation is quite long at 3.8 Å.

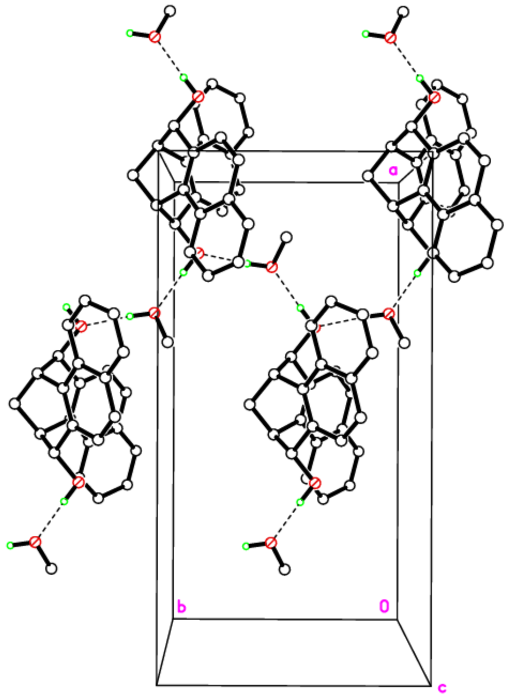

Figure S21. Packing plot for (±)-**7*a*** showing the methanol molecules bridging cleft molecules via H-bonds in a zig-zag chain parallel to *b*.

Table S1. Experimental details for (±)-***5a***

Crystal data	
Chemical formula	$C_{25}H_{16}O_2$
M_r	348.38
Crystal system, space group	Orthorhombic, *Pna*2_1
Temperature (K)	150(2)
a, *b*, *c* (Å)	10.3353 (10), 14.2207 (14), 11.2705 (11)
V (Å^3)	1656.5 (3)
Z	4
Radiation type	Mo *K*α
μ (mm^{-1})	0.09
Crystal size (mm^3)	0.70 × 0.18 × 0.09
Data collection	
Diffractometer	Bruker *APEX* 2 CCD area detector diffractometer
Absorption correction	Multi-scan<br>*SADABS* v2016/2, Sheldrick, G.M., (2014)
T_{min}, T_{max}	0.941, 0.992
No. of measured, independent and observed [$I > 2\sigma(I)$] reflections	17993, 4660, 4053
R_{int}	0.033
$(\sin \theta/\lambda)_{max}$ (Å^{-1})	0.696
Refinement	
$R[F^2 > 2\sigma(F^2)]$, $wR(F^2)$, S	0.039, 0.097, 1.07
No. of reflections	4660
No. of parameters	308
No. of restraints	1
H-atom treatment	All H-atom parameters refined
$\Delta\rangle_{max}$, $\Delta\rangle_{min}$ (e Å^{-3})	0.25, –0.18
Absolute structure	Flack x determined using 1704 quotients [(I+)-(I-)]/[(I+)+(I-)]. (Parsons, Flack and Wagner, Acta Cryst. B69 (2013) 249-259).
Absolute structure parameter	0.3 (5) {not reliably determined}

Computer programs: Bruker *APEX* 2, Bruker *SAINT*, SHELXT 2014/5 (Sheldrick, 2014), *SHELXL2018*/3 (Sheldrick, 2018), Bruker *SHELXTL*.

Table S2. Experimental details for (±)-***5b***

Crystal data	
Chemical formula	$C_{25}H_{16}O_2$
M_r	348.38
Crystal system, space group	Tetragonal, $P4_2/ncm$
Temperature (K)	150
a, *c* (Å)	12.0230 (12), 11.7827 (16)
V (Å^3)	1703.2 (4)
Z	4
Radiation type	Mo *K*α
μ (mm^{-1})	0.09
Crystal size (mm^3)	0.49 × 0.26 × 0.06
Data collection	
Diffractometer	Bruker *APEX* 2 CCD area detector diffractometer
Absorption correction	Multi-scan<br>*SADABS* v2016/2, Sheldrick, G.M., (2014)
T_{min}, T_{max}	0.959, 0.995
No. of measured, independent and observed [$I > 2\sigma(I)$] reflections	10993, 930, 680
R_{int}	0.030
$(\sin \theta/\lambda)_{max}$ (Å^{-1})	0.624
Refinement	
$R[F^2 > 2\sigma(F^2)]$, $wR(F^2)$, S	0.152, 0.483, 1.15
No. of reflections	930
No. of parameters	86
No. of restraints	187
H-atom treatment	H atoms treated by a mixture of independent and constrained refinement
$\Delta\rangle_{max}$, $\Delta\rangle_{min}$ (e Å^{-3})	0.75, –0.58

Computer programs: Bruker *APEX* 3, Bruker *SAINT*, SHELXT (Sheldrick, 2015), *SHELXL2018*/3 (Sheldrick, 2018), Bruker *SHELXTL*.

Table S3. Experimental details for (±)-***5c***

Crystal data	
Chemical formula	$C_{25}H_{16}O_2$
M_r	348.38
Crystal system, space group	Orthorhombic, *Pccn*
Temperature (K)	100
a, *b*, *c* (Å)	12.9727 (2), 10.78355 (17), 11.89717 (19)
V (Å^3)	1664.32 (5)
Z	4
Radiation type	Cu *K*α
μ (mm^{-1})	0.69
Crystal size (mm^3)	0.17 × 0.14 × 0.09
Data collection	
Diffractometer	Rigaku 007HF equipped with Varimax confocal mirrors and an AFC11 goniometer and HyPix 6000 detector
Absorption correction	Gaussian *CrysAlis PRO* 1.171.41.112a (Rigaku Oxford Diffraction, 2021). Numerical absorption correction based on gaussian integration over a multifaceted crystal model. Empirical absorption correction using spherical harmonics, implemented in SCALE3 ABSPACK scaling algorithm.
T_{min}, T_{max}	0.644, 1.000
No. of measured, independent and observed [$I > 2\sigma(I)$] reflections	27572, 1526, 1505
R_{int}	0.041
$(\sin \theta/\lambda)_{max}$ (Å^{-1})	0.602
Refinement	
$R[F^2 > 2\sigma(F^2)]$, $wR(F^2)$, S	0.034, 0.093, 1.04
No. of reflections	1526
No. of parameters	155
H-atom treatment	All H-atom parameters refined
$\Delta\rangle_{max}$, $\Delta\rangle_{min}$ (e Å^{-3})	0.30, -0.18

Computer programs: *CrysAlis PRO* 1.171.41.112a (Rigaku OD, 2021), SHELXT 2018/2 (Sheldrick, 2018), *SHELXL2018*/3 (Sheldrick, 2018), Bruker *SHELXTL*.

Table S4. Experimental details for (±)-***5d***

Crystal data	
Chemical formula	$C_{25}H_{16}O_2$
M_r	348.38
Crystal system, space group	Orthorhombic, $Pna2_1$
Temperature (K)	100
a, *b*, *c* (Å)	11.8233 (5), 12.3688 (5), 11.5639 (4)
V (Å^3)	1691.11 (11)
Z	4
Radiation type	Cu *K*α
μ (mm^{-1})	0.68
Crystal size (mm^3)	0.12 × 0.04 × 0.04
Data collection	
Diffractometer	Rigaku 007HF equipped with Varimax confocal mirrors and an AFC11 goniometer and HyPix 6000 detector.
Absorption correction	Multi-scan. *CrysAlis PRO* 1.171.41.93a (Rigaku Oxford Diffraction, 2020). Empirical absorption correction using spherical harmonics, implemented in SCALE3 ABSPACK scaling algorithm.
T_{min}, T_{max}	0.723, 1.000
No. of measured, independent and observed [$I > 2\sigma(I)$] reflections	16914, 3054, 2376
R_{int}	0.047
(sin θ/λ)$_{max}$ (Å^{-1})	0.603
Refinement	
$R[F^2 > 2\sigma(F^2)]$, $wR(F^2)$, S	0.231, 0.562, 1.05
No. of reflections	3054
No. of parameters	245
No. of restraints	590
H-atom treatment	H-atom parameters constrained
$\Delta\rangle_{max}$, $\Delta\rangle_{min}$ (e Å^{-3})	1.21, -0.50
Absolute structure	Refined as an inversion twin.
Absolute structure parameter	0.5 (22)

Computer programs: *CrysAlis PRO* 1.171.41.93a (Rigaku OD, 2020), SHELXT-2018/2 (Sheldrick, 2015), *SHELXL2018*/3 (Sheldrick, 2018), Bruker *SHELXTL*.

Table S5. Experimental details for (±)-***7a***

Crystal data	
Chemical formula	$C_{25}H_{20}O_2·2(CH_4O)$
M_r	416.49
Crystal system, space group	Orthorhombic, *Pbcn*
Temperature (K)	150
a, *b*, *c* (Å)	16.8601 (11), 8.6418 (6), 14.3431 (10)
V (Å^3)	2089.8 (2)
Z	4
Radiation type	Mo *K*α
μ (mm^{-1})	0.09
Crystal size (mm^3)	0.36 × 0.25 × 0.22
Data collection	
Diffractometer	Bruker *APEX* 2 CCD area detector diffractometer
Absorption correction	Multi-scan<br>*SADABS* v2016/2, Sheldrick, G.M., (2014)
T_{min}, T_{max}	0.969, 0.981
No. of measured, independent and observed [$I > 2\sigma(I)$] reflections	23518, 3194, 2802
R_{int}	0.023
$(\sin \theta/\lambda)_{max}$ (Å^{-1})	0.715
Refinement	
$R[F^2 > 2\sigma(F^2)]$, $wR(F^2)$, S	0.040, 0.115, 1.05
No. of reflections	3194
No. of parameters	197
H-atom treatment	All H-atom parameters refined
$\Delta〉_{max}$, $\Delta〉_{min}$ (e Å^{-3})	0.45, -0.18

Computer programs: Bruker *APEX* 2, Bruker *SAINT*, SHELXT 2014/5 (Sheldrick, 2014), *SHELXL2018*/3 (Sheldrick, 2018), Bruker *SHELXTL*.

Calculations of the interplanar cleft angle

The interplanar angle of (±)-**5a** was determined from the single crystal X-ray data using the two aromatic rings directly attached to the saturated cleft.

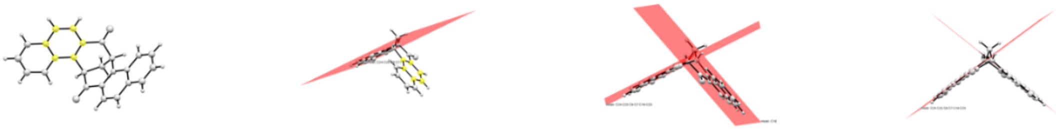

Step 1 *Step 2* *Step 3* *Step 4*

Figure S22. The plane of each aromatic ring was determined and then the angle between the two planes calculated using Mercury.[7]

A. (±)-**5a** interplanar angle of 105.2°

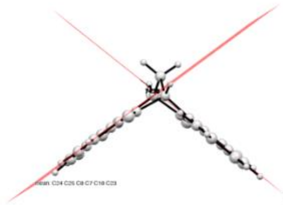

B. (±)-**5b** interplanar angle of 92.9°

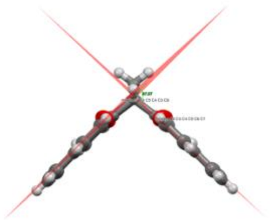

C. (±)-**5c** interplanar angle of 91.3°

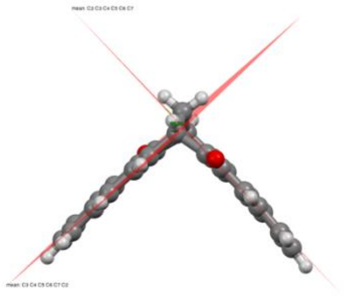

D. (±)-**5d** interplanar angle of 93.2°

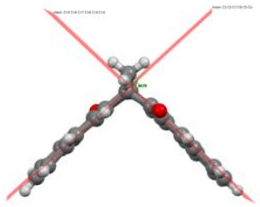

F. (±)-**7a** interplanar angle of 90.7°

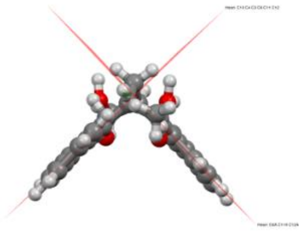

Figure S23. Calculated interplanar angles from X-ray data (±)-**5a-d** and (±)-**7a**.

Geometries of optimised structures

The structure of each carbocyclic cleft isomer was built with Gabedit[8] and optimized with *ab initio* Restrict Hartree-fock/6-31G(d) using Gaussian16.[9] The atomic partial charges were obtained according to Restrained Electrostatic Potential (RESP)[10] using the RESP ESP Charge Derive Server (R.E.D Server).[11]

Table S6. Molecular coordinates of (*R*,*R*)-**5a** optimized with *ab initio* Restrict Hartree-fock/6-31G(d).

Number	Label	Charge	SybylType	Xfrac + ESD	Yfrac + ESD	Zfrac + ESD	Symm. op.
1	C1	0	Du	0.0000	-0.0000	2.3905	Unknown
2	H1	0	Du	-0.5012	-0.7171	3.0329	Unknown
3	H2	0	Du	0.5012	0.7171	3.0329	Unknown
4	C2	0	Du	1.0140	-0.7080	1.4974	Unknown
5	H3	0	Du	1.7565	-1.2050	2.1043	Unknown
6	C3	0	C.2	0.2812	-1.7910	0.7149	Unknown
7	O1	0	O.3	0.7799	-2.8589	0.5160	Unknown
8	C4	0	Du	-1.0856	-1.4543	0.2171	Unknown
9	C5	0	Du	-1.6998	-0.2740	0.5500	Unknown
10	C6	0	Du	-1.0140	0.7080	1.4974	Unknown
11	H4	0	Du	-1.7565	1.2050	2.1043	Unknown
12	C7	0	C.2	-0.2812	1.7910	0.7149	Unknown
13	O	0	O.3	-0.7799	2.8589	0.5160	Unknown
14	C8	0	Du	1.0856	1.4543	0.2171	Unknown
15	C9	0	Du	1.6998	0.2740	0.5500	Unknown
16	C10	0	Du	2.9991	-0.0165	0.0146	Unknown
17	C11	0	Du	3.6322	0.9454	-0.8023	Unknown
18	C12	0	Ca	2.9653	2.1645	-1.1030	Unknown
19	H5	0	Du	3.4589	2.8855	-1.7300	Unknown
20	C13	0	Ca	1.7294	2.4077	-0.6168	Unknown
21	H6	0	Du	1.2087	3.3182	-0.8410	Unknown
22	C14	0	Ca	4.9260	0.6811	-1.3184	Unknown
23	H7	0	Du	5.3980	1.4270	-1.9329	Unknown
24	C15	0	Ca	5.5577	-0.4905	-1.0474	Unknown
25	H8	0	Du	6.5383	-0.6830	-1.4444	Unknown
26	C16	0	Ca	4.9214	-1.4665	-0.2493	Unknown
27	H9	0	Du	5.4178	-2.3999	-0.0545	Unknown
28	C17	0	Ca	3.6833	-1.2392	0.2649	Unknown
29	H10	0	Du	3.2084	-2.0109	0.8359	Unknown
30	C18	0	Du	-2.9991	0.0165	0.0146	Unknown
31	C19	0	Du	-3.6322	-0.9454	-0.8023	Unknown
32	C20	0	Ca	-2.9653	-2.1645	-1.1030	Unknown
33	H11	0	Du	-3.4589	-2.8855	-1.7300	Unknown
34	C21	0	Ca	-1.7294	-2.4077	-0.6168	Unknown
35	H12	0	Du	-1.2087	-3.3182	-0.8410	Unknown
36	C22	0	Ca	-4.9260	-0.6811	-1.3184	Unknown
37	H13	0	Du	-5.3980	-1.4270	-1.9329	Unknown
38	C23	0	Ca	-5.5577	0.4905	-1.0474	Unknown
39	H14	0	Du	-6.5383	0.6830	-1.4444	Unknown
40	C24	0	Ca	-4.9214	1.4665	-0.2493	Unknown
41	H15	0	Du	-5.4178	2.3999	-0.0545	Unknown
42	C	0	Ca	-3.6833	1.2392	0.2649	Unknown
43	H	0	Du	-3.2084	2.0109	0.8359	Unknown

Table S7. Molecular coordinates of (*S*,*S*)-**5a** optimized with *ab initio* Restrict Hartree-fock/6-31G(d).

Number	Label	Charge	SybylType	Xfrac + ESD	Yfrac + ESD	Zfrac + ESD	Symm. op.
1	C1	0	Du	-0.0000	-0.0000	2.3905	Unknown
2	H1	0	Du	0.5012	-0.7171	3.0329	Unknown
3	H2	0	Du	-0.5012	0.7171	3.0329	Unknown
4	C2	0	Du	1.0140	0.7080	1.4974	Unknown
5	H3	0	Du	1.7565	1.2050	2.1043	Unknown
6	C3	0	Du	1.6998	-0.2740	0.5500	Unknown
7	C4	0	Du	1.0856	-1.4543	0.2171	Unknown
8	C5	0	C.2	-0.2812	-1.7910	0.7149	Unknown
9	O1	0	O.3	-0.7799	-2.8589	0.5160	Unknown
10	C6	0	Du	-1.0140	-0.7080	1.4974	Unknown
11	H4	0	Du	-1.7565	-1.2050	2.1043	Unknown
12	C7	0	Du	-1.6998	0.2740	0.5500	Unknown
13	C8	0	Du	-1.0856	1.4543	0.2171	Unknown
14	C9	0	C.2	0.2812	1.7910	0.7149	Unknown
15	O	0	O.3	0.7799	2.8589	0.5160	Unknown
16	C10	0	Ca	-1.7294	2.4077	-0.6168	Unknown
17	H5	0	Du	-1.2087	3.3182	-0.8410	Unknown
18	C11	0	Ca	-2.9653	2.1645	-1.1030	Unknown
19	H6	0	Du	-3.4589	2.8855	-1.7300	Unknown
20	C12	0	Du	-3.6322	0.9454	-0.8023	Unknown
21	C13	0	Du	-2.9991	-0.0165	0.0146	Unknown
22	C14	0	Ca	-3.6833	-1.2392	0.2649	Unknown
23	H7	0	Du	-3.2084	-2.0109	0.8359	Unknown
24	C15	0	Ca	-4.9214	-1.4665	-0.2492	Unknown
25	H8	0	Du	-5.4178	-2.3998	-0.0545	Unknown
26	C16	0	Ca	-5.5578	-0.4905	-1.0474	Unknown
27	H9	0	Du	-6.5383	-0.6830	-1.4444	Unknown
28	C17	0	Ca	-4.9260	0.6811	-1.3183	Unknown
29	H10	0	Du	-5.3980	1.4270	-1.9329	Unknown
30	C18	0	Ca	1.7294	-2.4077	-0.6168	Unknown
31	H11	0	Du	1.2087	-3.3182	-0.8410	Unknown
32	C19	0	Ca	2.9653	-2.1645	-1.1030	Unknown
33	H12	0	Du	3.4589	-2.8855	-1.7300	Unknown
34	C20	0	Du	3.6322	-0.9454	-0.8023	Unknown
35	C21	0	Du	2.9991	0.0165	0.0146	Unknown
36	C22	0	Ca	3.6833	1.2392	0.2649	Unknown
37	H13	0	Du	3.2084	2.0109	0.8359	Unknown
38	C23	0	Ca	4.9214	1.4665	-0.2492	Unknown
39	H14	0	Du	5.4178	2.3998	-0.0545	Unknown
40	C24	0	Ca	5.5578	0.4905	-1.0474	Unknown
41	H15	0	Du	6.5383	0.6830	-1.4444	Unknown
42	C	0	Ca	4.9260	-0.6811	-1.3183	Unknown
43	H	0	Du	5.3980	-1.4270	-1.9329	Unknown

Table S8. Molecular coordinates of (*R*,*R*)-**5b** optimized with *ab initio* Restrict Hartree-fock/6-31G(d).

Number	Label	Charge	SybylType	Xfrac + ESD	Yfrac + ESD	Zfrac + ESD	Symm. op.
1	C1	0	Du	-0.0000	0.0000	2.8603	Unknown
2	H1	0	Du	0.8710	0.0961	3.5009	Unknown
3	H2	0	Du	-0.8710	-0.0961	3.5009	Unknown
4	C2	0	Du	-0.1371	1.2028	1.9456	Unknown
5	H3	0	Du	-0.2738	2.1157	2.5094	Unknown
6	C3	0	C.2	1.1550	1.4024	1.1518	Unknown
7	O1	0	O.3	1.5695	2.5146	1.0046	Unknown
8	C4	0	Du	1.8122	0.1728	0.6022	Unknown
9	C5	0	Du	1.3080	-1.0497	0.9831	Unknown
10	C6	0	Du	0.1371	-1.2028	1.9456	Unknown
11	H4	0	Du	0.2738	-2.1157	2.5094	Unknown
12	C7	0	C.2	-1.1550	-1.4024	1.1518	Unknown
13	O	0	O.3	-1.5695	-2.5145	1.0046	Unknown
14	C8	0	Du	-1.8122	-0.1728	0.6022	Unknown
15	C9	0	Du	-1.3080	1.0497	0.9831	Unknown
16	C10	0	Ca	-1.8645	2.2486	0.4722	Unknown
17	H5	0	Du	-1.4400	3.1879	0.7753	Unknown
18	C11	0	Ca	-2.9071	2.2089	-0.3877	Unknown
19	H6	0	Du	-3.3271	3.1222	-0.7709	Unknown
20	C12	0	Du	-3.4689	0.9737	-0.8007	Unknown
21	C13	0	Du	-2.9301	-0.2367	-0.3123	Unknown
22	C14	0	Ca	-3.5260	-1.4473	-0.7672	Unknown
23	H7	0	Du	-3.1470	-2.3816	-0.4190	Unknown
24	C15	0	Ca	-4.5746	-1.4282	-1.6356	Unknown
25	H8	0	Du	-5.0041	-2.3586	-1.9616	Unknown
26	C16	0	Ca	-5.1080	-0.2134	-2.1146	Unknown
27	H9	0	Du	-5.9368	-0.2220	-2.7994	Unknown
28	C17	0	Ca	-4.5626	0.9591	-1.7028	Unknown
29	H10	0	Du	-4.9504	1.8984	-2.0560	Unknown
30	C18	0	Ca	1.8645	-2.2486	0.4722	Unknown
31	H11	0	Du	1.4400	-3.1879	0.7753	Unknown
32	C19	0	Ca	2.9071	-2.2089	-0.3877	Unknown
33	H12	0	Du	3.3271	-3.1222	-0.7709	Unknown
34	C20	0	Du	3.4689	-0.9737	-0.8007	Unknown
35	C21	0	Du	2.9301	0.2367	-0.3123	Unknown
36	C22	0	Ca	3.5260	1.4473	-0.7672	Unknown
37	H13	0	Du	3.1470	2.3816	-0.4190	Unknown
38	C23	0	Ca	4.5746	1.4282	-1.6356	Unknown
39	H14	0	Du	5.0041	2.3586	-1.9616	Unknown
40	C24	0	Ca	5.1080	0.2133	-2.1146	Unknown
41	H15	0	Du	5.9368	0.2220	-2.7994	Unknown
42	C	0	Ca	4.5626	-0.9591	-1.7028	Unknown
43	H	0	Du	4.9504	-1.8985	-2.0560	Unknown

Table S9. Molecular coordinates of (*S*,*S*)-**5b** optimized with *ab initio* Restrict Hartree-fock/6-31G(d).

Number	Label	Charge	SybylType	Xfrac + ESD	Yfrac + ESD	Zfrac + ESD	Symm. op.
1	C1	0	Du	0.0000	-0.0000	2.8602	Unknown
2	H1	0	Du	0.8710	-0.0961	3.5009	Unknown
3	H2	0	Du	-0.8710	0.0961	3.5009	Unknown
4	C2	0	Du	0.1371	1.2028	1.9456	Unknown
5	H3	0	Du	0.2738	2.1157	2.5094	Unknown
6	C3	0	Du	1.3080	1.0497	0.9831	Unknown
7	C4	0	Du	1.8122	-0.1728	0.6022	Unknown
8	C5	0	C.2	1.1550	-1.4024	1.1518	Unknown
9	O1	0	O.3	1.5695	-2.5146	1.0045	Unknown
10	C6	0	Du	-0.1371	-1.2028	1.9456	Unknown
11	H4	0	Du	-0.2738	-2.1157	2.5094	Unknown
12	C7	0	Du	-1.3080	-1.0497	0.9831	Unknown
13	C8	0	Du	-1.8122	0.1728	0.6022	Unknown
14	C9	0	C.2	-1.1550	1.4024	1.1518	Unknown
15	O	0	O.3	-1.5695	2.5145	1.0046	Unknown
16	C10	0	Du	-2.9301	0.2367	-0.3123	Unknown
17	C11	0	Du	-3.4689	-0.9737	-0.8007	Unknown
18	C12	0	Ca	-2.9071	-2.2089	-0.3877	Unknown
19	H5	0	Du	-3.3271	-3.1222	-0.7709	Unknown
20	C13	0	Ca	-1.8645	-2.2486	0.4722	Unknown
21	H6	0	Du	-1.4401	-3.1879	0.7753	Unknown
22	C14	0	Ca	-4.5627	-0.9591	-1.7028	Unknown
23	H7	0	Du	-4.9505	-1.8984	-2.0560	Unknown
24	C15	0	Ca	-5.1080	0.2134	-2.1146	Unknown
25	H8	0	Du	-5.9368	0.2221	-2.7994	Unknown
26	C16	0	Ca	-4.5746	1.4282	-1.6356	Unknown
27	H9	0	Du	-5.0041	2.3587	-1.9616	Unknown
28	C17	0	Ca	-3.5260	1.4473	-0.7672	Unknown
29	H10	0	Du	-3.1470	2.3816	-0.4190	Unknown
30	C18	0	Du	2.9301	-0.2367	-0.3123	Unknown
31	C19	0	Du	3.4689	0.9737	-0.8007	Unknown
32	C20	0	Ca	2.9071	2.2089	-0.3877	Unknown
33	H11	0	Du	3.3271	3.1222	-0.7709	Unknown
34	C21	0	Ca	1.8645	2.2486	0.4722	Unknown
35	H12	0	Du	1.4401	3.1879	0.7753	Unknown
36	C22	0	Ca	4.5627	0.9591	-1.7028	Unknown
37	H13	0	Du	4.9505	1.8985	-2.0560	Unknown
38	C23	0	Ca	5.1080	-0.2134	-2.1146	Unknown
39	H14	0	Du	5.9368	-0.2220	-2.7993	Unknown
40	C24	0	Ca	4.5746	-1.4282	-1.6356	Unknown
41	H15	0	Du	5.0042	-2.3586	-1.9616	Unknown
42	C	0	Ca	3.5260	-1.4473	-0.7672	Unknown
43	H	0	Du	3.1470	-2.3816	-0.4189	Unknown

Table S10. Molecular coordinates of **5c** optimized with *ab initio* Restrict Hartree-fock/6-31G(d).

Number	Label	Charge	SybylType	Xfrac + ESD	Yfrac + ESD	Zfrac + ESD	Symm. op.
1	C1	0	Du	-0.0000	-0.0000	2.8286	Unknown
2	H1	0	Du	0.7491	-0.4509	3.4724	Unknown
3	H2	0	Du	-0.7491	0.4509	3.4724	Unknown
4	C2	0	Du	0.6465	1.0609	1.9366	Unknown
5	H3	0	Du	1.1316	1.8252	2.5294	Unknown
6	C3	0	Du	1.6708	0.4346	0.9991	Unknown
7	C4	0	Du	1.5498	-0.9328	0.6113	Unknown
8	C5	0	C.2	0.4207	-1.7719	1.1112	Unknown
9	O1	0	O.3	0.3377	-2.9402	0.8740	Unknown
10	C6	0	Du	-0.6465	-1.0609	1.9366	Unknown
11	H4	0	Du	-1.1316	-1.8252	2.5294	Unknown
12	C7	0	Du	-1.6708	-0.4346	0.9991	Unknown
13	C8	0	Du	-1.5498	0.9327	0.6113	Unknown
14	C9	0	C.2	-0.4207	1.7719	1.1112	Unknown
15	O	0	O.3	-0.3377	2.9402	0.8740	Unknown
16	C10	0	Ca	-2.4702	1.4947	-0.2253	Unknown
17	H5	0	Du	-2.3594	2.5276	-0.4987	Unknown
18	C11	0	Du	-3.5527	0.7435	-0.7343	Unknown
19	C12	0	Du	-3.6706	-0.6103	-0.3603	Unknown
20	C13	0	Ca	-2.7066	-1.1723	0.5144	Unknown
21	H6	0	Du	-2.8023	-2.2067	0.7940	Unknown
22	C14	0	Ca	-4.7573	-1.3705	-0.8707	Unknown
23	H7	0	Du	-4.8480	-2.4042	-0.5878	Unknown
24	C15	0	Ca	-5.6656	-0.8017	-1.7045	Unknown
25	H8	0	Du	-6.4842	-1.3843	-2.0874	Unknown
26	C16	0	Ca	-5.5472	0.5607	-2.0805	Unknown
27	H9	0	Du	-6.2767	0.9925	-2.7415	Unknown
28	C17	0	Ca	-4.5198	1.3109	-1.6085	Unknown
29	H10	0	Du	-4.4224	2.3447	-1.8891	Unknown
30	C18	0	Ca	2.4702	-1.4947	-0.2253	Unknown
31	H11	0	Du	2.3593	-2.5276	-0.4987	Unknown
32	C19	0	Du	3.5527	-0.7435	-0.7343	Unknown
33	C20	0	Du	3.6706	0.6103	-0.3603	Unknown
34	C21	0	Ca	2.7066	1.1723	0.5144	Unknown
35	H12	0	Du	2.8023	2.2067	0.7941	Unknown
36	C22	0	Ca	4.7573	1.3705	-0.8707	Unknown
37	H13	0	Du	4.8480	2.4042	-0.5878	Unknown
38	C23	0	Ca	5.6656	0.8017	-1.7045	Unknown
39	H14	0	Du	6.4842	1.3843	-2.0874	Unknown
40	C24	0	Ca	5.5472	-0.5607	-2.0805	Unknown
41	H15	0	Du	6.2767	-0.9925	-2.7415	Unknown
42	C	0	Ca	4.5198	-1.3108	-1.6085	Unknown
43	H	0	Du	4.4224	-2.3447	-1.8891	Unknown

Table S11. Molecular coordinates of **5d** optimized with *ab initio* Restrict Hartree-fock/6-31G(d).

Number	Label	Charge	SybylType	Xfrac + ESD	Yfrac + ESD	Zfrac + ESD	Symm. op.
1	C1	0	Du	0.1657	1.8915	2.1684	Unknown
2	H1	0	Du	-0.6514	2.5072	2.5323	Unknown
3	H2	0	Du	0.9877	1.9934	2.8699	Unknown
4	C2	0	Du	-0.2679	0.4352	2.0596	Unknown
5	H3	0	Du	-0.5875	0.0485	3.0177	Unknown
6	C3	0	Du	-1.4027	0.2740	1.0577	Unknown
7	C4	0	Du	-1.5525	1.2048	-0.0128	Unknown
8	C5	0	C.2	-0.5907	2.3340	-0.1714	Unknown
9	O1	0	O.3	-0.7240	3.1840	-1.0003	Unknown
10	C6	0	Du	0.6031	2.3372	0.7795	Unknown
11	H4	0	Du	0.9826	3.3506	0.7927	Unknown
12	C7	0	Du	1.6793	1.4226	0.2064	Unknown
13	C8	0	Du	1.8471	0.1151	0.6022	Unknown
14	C9	0	C.2	0.9222	-0.4312	1.6495	Unknown
15	O	0	O.3	1.0591	-1.4959	2.1768	Unknown
16	C10	0	Du	2.8909	-0.6876	0.0023	Unknown
17	C11	0	Du	3.7080	-0.0991	-0.9876	Unknown
18	C12	0	Ca	3.4888	1.2501	-1.3679	Unknown
19	H5	0	Du	4.1182	1.6832	-2.1252	Unknown
20	C13	0	Ca	2.5100	1.9852	-0.7953	Unknown
21	H6	0	Du	2.3431	3.0032	-1.0953	Unknown
22	C14	0	Ca	4.7397	-0.8494	-1.6058	Unknown
23	H7	0	Du	5.3450	-0.3708	-2.3554	Unknown
24	C15	0	Ca	4.9583	-2.1443	-1.2628	Unknown
25	H8	0	Du	5.7407	-2.7117	-1.7337	Unknown
26	C16	0	Ca	4.1446	-2.7435	-0.2792	Unknown
27	H9	0	Du	4.3140	-3.7700	-0.0068	Unknown
28	C17	0	Ca	3.1475	-2.0482	0.3348	Unknown
29	H10	0	Du	2.5522	-2.5275	1.0785	Unknown
30	C18	0	Ca	-2.5749	1.0713	-0.9058	Unknown
31	H11	0	Du	-2.6676	1.7883	-1.7004	Unknown
32	C19	0	Du	-3.5016	0.0093	-0.8029	Unknown
33	C20	0	Du	-3.3525	-0.9180	0.2479	Unknown
34	C21	0	Ca	-2.2856	-0.7549	1.1687	Unknown
35	H12	0	Du	-2.1796	-1.4661	1.9689	Unknown
36	C22	0	Ca	-4.2809	-1.9878	0.3548	Unknown
37	H13	0	Du	-4.1680	-2.6974	1.1551	Unknown
38	C23	0	Ca	-5.2940	-2.1145	-0.5409	Unknown
39	H14	0	Du	-5.9916	-2.9280	-0.4535	Unknown
40	C24	0	Ca	-5.4442	-1.1813	-1.5978	Unknown
41	H15	0	Du	-6.2527	-1.2987	-2.2965	Unknown
42	C	0	Ca	-4.5722	-0.1492	-1.7244	Unknown
43	H	0	Du	-4.6787	0.5628	-2.5236	Unknown

References

(1) H. Tatemitsu, F. Ogura, Y. Nakagawa, M. Nakagawa, K. Naemura and M. Nakazaki, *Bull. Chem. Soc. Jap.* 1975, **48**, 2473.

(2) K. Naemura and R. Fukunaga, *Chem. Lett.* 1985, 1651.

(3) SAINT and APEX 2 (2014) software for CCD diffractometers. Bruker AXS Inc., Madison, USA.

(4) CrysAlis Pro, (2021), Rigaku Corp.

(5) Sheldrick, G. M. *Acta Cryst*. 2015, **A71,** 3-8. (XT)

(6) Sheldrick, G. M. *Acta Cryst*. 2015, **C71,** 3-8. (XL)

(7) Mercury 4.0: from visualisation to analysis, design and prediction. Macrae, C. F; Sovago, I.; Cottrell, S. J.; Galek, P. T. A.; McCabe, P.; Pidcock, E.; Platings, M.; Shields, G. P.; Stevens, J. S.; Towler, M.; Wood, P. A. *J. Appl. Cryst.* 2020, **53**, 2020.

(8) Allouche, A. R. *J. Comp. Chem.* 2011, **32**, 174.

(9) M. J. Frisch, G. W. Trucks, H. B. Schlegel, G. E. Scuseria, M. A. Robb, J. R. Cheeseman, G. Scalmani, V. Barone, G. A. Petersson, H. Nakatsuji, X. Li, M. Caricato, A. V. Marenich, J. Bloino, B. G. Janesko, R. Gomperts, B. Mennucci, H. P. Hratchian, J. V. Ortiz, A. F. Izmaylov, J. L. Sonnenberg, D. Williams-Young, F. Ding, F. Lipparini, F. Egidi, J. Goings, B. Peng, A. Petrone, T. Henderson, D. Ranasinghe, V. G. Zakrzewski, J. Gao, N. Rega, G. Zheng, W. Liang, M. Hada, M. Ehara, K. Toyota, R. Fukuda, J.Hasegawa, M. Ishida, T. Nakajima, Y. Honda, O. Kitao, H. Nakai, T. Vreven, K. Throssell, J. A.Montgomery, Jr., J. E. Peralta, F. Ogliaro, M. J. Bearpark, J. J. Heyd, E. N. Brothers, K. N. Kudin, V. N.Staroverov, T. A. Keith, R. Kobayashi, J. Normand, K. Raghavachari, A. P. Rendell, J. C. Burant, S. S.Iyengar, J. Tomasi, M. Cossi, J. M. Millam, M. Klene, C. Adamo, R. Cammi, J. W. Ochterski, R. L.Martin, K. Morokuma, O. Farkas, J. B. Foresman, and D. J. Fox, Gaussian, Inc., Wallingford CT, 2016

(10) Bayly, C. I.; Cieplak, P.; Cornell, W.; Kollman, P. A. *J. Phys. Chem.* 1993, **97**, 10269.

(11) Vanquelef, E.; Simon, S.; Marquant, G.; Garcia, E.; Kilmerak, G.; Delepine, J. C.; Cieplak, P.; Dupradeau, F. Y. *Nucl. Acid Res.* 2011, **39**, W511-W517.
